# Hyperosmotic stress activates the expression of members of the miR-15/107 family and induces downregulation of anti-apoptotic genes in rat liver

**DOI:** 10.1038/srep12292

**Published:** 2015-07-21

**Authors:** David Santosa, Mirco Castoldi, Martha Paluschinski, Annika Sommerfeld, Dieter Häussinger

**Affiliations:** 1 Department of Gastroenterology, Hepatology and Infectious Diseases, Heinrich-Heine-University, Moorenstrasse 5, 40225 Düsseldorf, Germany

## Abstract

microRNAs are an abundant class of small non-coding RNAs that negatively regulate gene expression. Importantly, microRNA activity has been linked to the control of cellular stress response. In the present study, we investigated whether the expression of hepatic microRNAs is affected by changes in ambient osmolarity. It is shown that hyperosmotic exposure of perfused rat liver induces a rapid upregulation of miR-15a, miR-15b and miR-16, which are members of the miR-15/107 microRNAs superfamily. It was also identified that hyperosmolarity significantly reduces the expression of anti-apoptotic genes including Bcl2, Ccnd1, Mcl1, Faim, Aatf, Bfar and Ikbkb, which are either validated or predicted targets of these microRNAs. Moreover, through the application of NOX and JNK inhibitors as well as benzylamine it is shown that the observed response is mediated by reactive oxygen species (ROS), suggesting that miR-15a, miR-15b and miR-16 are novel redoximiRs. It is concluded that the response of these three microRNAs to osmotic stress is ROS-mediated and that it might contribute to the development of a proapoptotic phenotype.

Several physiological functions depend on cell volume, which is not only influenced by ambient osmolarity, but can change within minutes also in response to hormones, cumulative substrate transport, bile acids or oxidative stress (for reviews see^1^,^2^). Several osmosensing and osmosignaling pathways have been identified, which couple cell volume to cell metabolism, transport, gene expression, proliferation and apoptosis (for review see^3^). For example, cell swelling and shrinkage result in the activation of catabolic or anabolic signaling^1^,^2^ respectively. Cell shrinkage, either induced by the exposure to hyperosmotic environment or to hydrophobic bile acids, can activate on a short-term level the CD95 death receptor in hepatocytes and sensitize to hepatocyte death^4^,^5^,^6^. microRNAs (miRNAs) are 22 nts long single stranded non-coding RNAs that inhibit gene expression at the posttranscriptional level. miRNA activity regulates diverse processes such as systemic iron homeostasis^7^,^8^, cell proliferation^9^, apoptosis^10^,^11^, reactive oxygen species formation (ROS;^12^,^13^) and safeguarding from environmental stress^14^. Importantly, dysregulation of miRNA expression has been linked to the biogenesis of several human diseases including but not limited to cancer^15^,^16^, diabetes^17^,^18^ and cholangiopathies^19^. Moreover, miRNAs could potentially play a role in the maintenance of osmotic homeostasis as it was shown that miRNAs participate in the regulation of osmotic stress in zebrafish^20^ and that aquaporin-1 expression is regulated by the osmotically sensitive miRNAs miR-666 and -708 in mice^21^.

Our data show that hyperosmolarity upregulates the expression of miR-15a, -15b and -16 and downregulates the expression of B-cell lymphoma 2 (Bcl2), cyclin D1 (Ccnd1), myeloid cell leukemia sequence 1 (Mcl1), Fas apoptotic inhibitory molecule (Faim), apoptosis antagonizing transcription factor (Aatf), bifunctional apoptosis regulator (Bfar) and inhibitor of kappa light polypeptide gene enhancer in B-cells, kinase beta (Ikbkb, IKK-β). This response is modulated by hyperosmolarity-induced oxidative stress through activation of NADPH-oxidase (NOX;^4^,^5^), a major source of ROS, and is attenuated by inhibition of NOX or of c-Jun-N-terminal kinase (JNK). Administration of benzylamine, which generates H_2_O_2_ via its metabolism at monoamine oxidase had similar effects.

## Results

### The expression of members of the miR-15 family is upregulated in response to hyperosmotic stimulation

**Figure 1:**
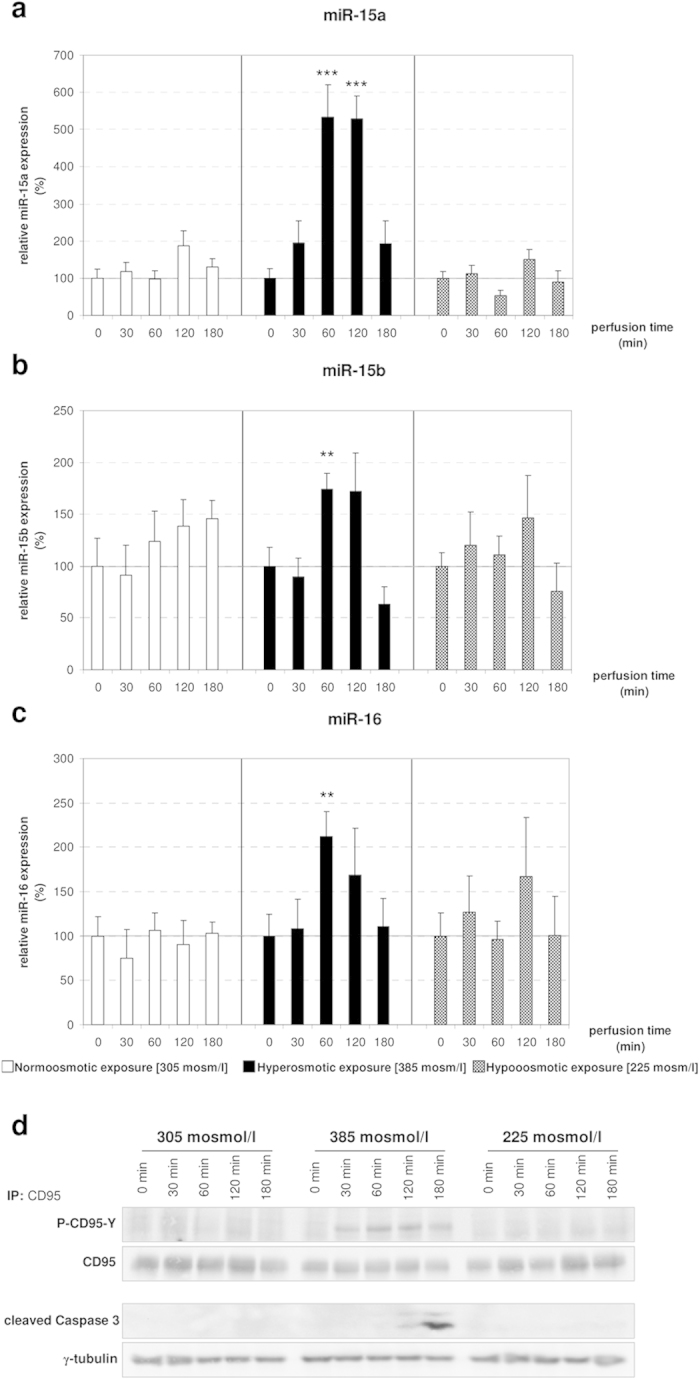
Upregulation of miR-15a/b and miR-16 by hyperosmolarity in perfused rat liver. Rat livers were perfused with normoosmotic medium (305 mosmol/l), hyperosmotic medium (385 mosmol/l) or hypoosmotic medium (225 mosmol) for up to 180 min. Samples were taken at the time points indicated. **(a)** miR-15a is significantly upregulated after 60 and 120 minutes of hyperosmotic conditions, whereas a stable expression is observed under hypo- and normoosmotic (305 mosmol/l) conditions (* p < 0.05; **p < 0.01; ***p < 0.001). **(b)** miR-15b is significantly upregulated at 60 minutes of hyperosmotic perfusion, while it is stably expressed under normoosmotic and hypoosmotic conditions. **(c)** miR-16 is significantly upregulated under hyperosmolarity. Statistical analysis was carried out by unpaired student’s t-test. Data are shown as average ± S.E.M. of 5 independent experiments. The values of unstimulated controls (T0) were set arbitrarily to 100**. (d)** CD95 was immunoprecipitated and activating CD95-tyrosine phosphorylation (P-CD95-Y) and caspase 3 cleavage were analysed by Western blot using specific antibodies. Total CD95 and γ-tubulin served as respective loading controls. Representative immunoblots from 3 independent experiments are depicted. Hyperosmolarity-induced activation of the CD95 was already detected after 30 min, whereas cleavage of caspase 3 occurred 120 min after exposure to hyperosmolarity. Normoosmotic- and hypoosmotic perfusion of rat liver do not lead to initiation of the apoptotic signaling pathway.

With the purpose to identify hepatic miRNAs, which are regulated in response to osmotic changes, rat livers were perfused with normo- (305 mosm/l), hyper- (385 mosm/l) or hypoosmotic (225 mosm/l) solutions. Following RNA isolation, the levels of miRNAs were measured by miQPCR as described in the Methods section. We found that the expression of miR-15a, miR-15b and miR-16 was significantly upregulated following perfusion of rat livers with hyperosmotic solution, whereas hypoosmotic perfusion had no significant effect on the expression level of these microRNAs (Fig. 1a-c). Specifically, miR-15a was found to be significantly upregulated at both 60 minutes (5.3-folds, p = 0.0002) and 120 minutes (5.3-folds; p = 0.0001) compared to the preperfusion state (defined as T0), while miR-15b (1.7-folds; p = 0.006) and miR-16 (2.1-folds; p = 0.0078) were both found significantly upregulated at 60 minutes after perfusion of the livers with hyperosmotic solution. Notably, these miRNAs belong to the miR-15/107 family, which comprises 10 paralogous miRNAs sharing the same seed sequence AGCAGC^22^. Altogether, these findings indicate that the expression levels of miR-15a, -15b and -16 are rapidly modulated in response to hyperosmotic stress.

### miR-15a, -15b and -16 upregulation could contribute to cell death through the inhibition of anti-apoptotic genes

**Figure 2:**
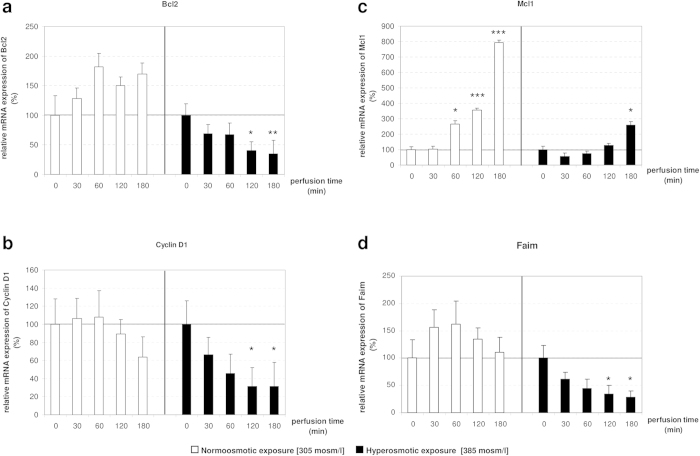
Target genes Bcl2, Mcl1, Ccnd1 and Faim are downregulated by hyperosmotic stimulation in perfused rat liver. mRNA analysis of target genes of the miR-15 family under hyper- and normoosmotic conditions in perfused rat liver. mRNA levels of hyper- and normoosmotically treated samples at specific time points (30, 60, 120 and 180 minutes) were compared to unstimulated controls (T0). **(a,b)** Bcl2 and Ccnd1 are significantly downregulated after 120 and 180 minutes of hyperosmotic perfusion. **(c)** Mcl1 is upregulated under normoosmotic conditions, while this effect is attenuated under hyperosmotic conditions. **(d)** The anti-apoptotic molecule Faim is significantly downregulated under hyperosmotic conditions and stably expressed in the normoosmotic control. qPCR runs were normalized according to the ΔΔCt method using Gapdh as reference gene. Statistical analysis was carried out by unpaired student’s t-test. Data are shown as average ± S.E.M. of 5 independent experiments. The values of unstimulated controls (T0) were set arbitrarily to 100.

Interestingly, miR-15a, -15b and -16 are known as tumor-suppressor miRNAs as their activity directly represses the expression of anti-apoptotic genes such as Bcl2 (validated in human, mouse and rat^23^,^24^), Ccnd1 (validated in human, mouse and rat^25^) and protein carboxyl-O-methyltransferase (Pcmt1; validated in human^26^). Mcl1 is indicated in the literature as validated miR-15a target in humans, however to the best of our knowledge, this interaction has never been experimentally validated and, for the purpose of this study, Mcl1 is included as miR-15a predicted target^27^. In order to evaluate whether the upregulation of these miRNAs results in the downregulation of their validated and predicted targets, qPCR was used to measure mRNA levels of Bcl2, Ccnd1, Mcl1 and Pcmt1 (Fig. 2 and Supplementary Figure 1). Interestingly, Bcl2 [0.4-fold after 120 minutes (p = 0.0127), 0.35-fold after 180 minutes (p = 0.0065)] and Ccnd1 [0.3-fold after 120 minutes (p = 0.0199), 0.3-fold after 180 minutes (p = 0.0227)] were found significantly downregulated by hyperosmotic stimulation (Fig. 2a-b). Whereas, Mcl1 expression shows a tendency toward downregulation compared to the control, although without reaching significance (Fig. 2c). Overall, these findings implicate that signaling pathways activated by hyperosmotic hepatocyte shrinkage downregulate the expression of anti-apoptotic genes, which are either validated (Bcl2 and Ccnd1) or predicted (Mcl1) miR-15/16 targets. Based on these data, we hypothesized that hyperosmolarity-mediated upregulation of miRNA expression could trigger or contribute to the downregulation of anti-apoptotic genes (Fig. 2).

Next, we examined whether the observed upregulation of miR-15a, -15b and -16 was sufficient to downregulate their targets at protein level. For this purpose Western Blot analysis was performed to quantify the amount of Bcl2 protein in the livers after hyper- and normoosmotic perfusion (Supplementary Fig. 2). Importantly, Bcl2 was selected as it was recently shown that miR-15a, -15b and -16 directly regulate the 3′UTR of rat Bcl2^24^. Altogether, our analysis shows that Bcl2 content does not differ between the livers of hyper- and normoosmotically perfused animals. Based on this data we concluded that the treatment period (i.e. three hours perfusion) may be too short to significantly impact the turnover of Bcl2 at protein level.

Recent studies on rat hepatocytes indicated that hyperosmotic cell shrinkage leads to an activation of the CD95 death receptor system, which involves CD95 tyrosine phosphorylation, CD95 oligomerization, and subsequent trafficking of the CD95 to the plasma membrane^4^,^5^,^6^. Mild hyperosmotic exposure (405 mosmol/l), does not lead to a reduction of cell viability, although DISC formation and subsequent caspase 8 and 3 activation occur, but sensitizes hepatocytes to CD95L-induced apoptosis^5^,^6^. In line with this, hyperosmolarity induced tyrosine phosphorylation of CD95 and subsequent caspase 3 activation (Fig. 1d). In addition, hyperosmolarity (405 mosm/l for 15 minutes) increased ROS formation 3.04 ± 0.44 fold (n = 3; p = 0.044) over control, as measured by CM-H_2_DCFDA fluorescence in primary hepatocytes.

### Fas apoptotic inhibitory molecule is a novel potential target of the miR-15 family

In order to identify additional putative targets of miR-15a, -15b and -16 that could be involved in regulation of cell death or survival we queried two different target prediction databases miRWalk and MicroCosm for miR-15b and miR-16 predicted targets. Reason for miR-15a exclusion from the query is that rno-miR-15a was only recently added to the miRNA repository database miRbase (miRbase v21, release June 2014), hence, to date no pre-compiled target predictions for miR-15a are available for the rat transcriptome. Importantly, the miRWalk algorithm performs the comparative analysis of the query against up to ten different target prediction databases, while the algorithm behind MicroCosm target prediction database looks for conservation of the putative miRNA binding site in at least two different species. Applying this approach, two putative binding sites for miR-15b and -16 were identified in the 3′UTR of Faim at positions 493/513 (rno-miR-16) and 292/313 (rno-miR-15b and rno-miR-16), suggesting that Faim could be a novel target for miR-15/16. Next, qPCR was used to evaluate whether Faim expression levels are inversely correlated with miR-15a, -15b and -16 expression in hyperosmotically perfused livers (Fig. 2d). Our analysis identified that Faim is significantly downregulated after 120 (0.35-fold change, p = 0.0491) and 180 (0.28-fold change, p = 0.0246) minutes of hyperosmotic perfusion. Altogether, these data show that Faim expression is inversely correlated to miR-15a/b and miR-16 expression, suggesting that Faim could be a novel functional target of miR-15a, -15b and -16.

### Apocynin leads to downregulation of the miR-15a and miR-16 in rat livers perfused with hyperosmotic solution

**Figure 3:**
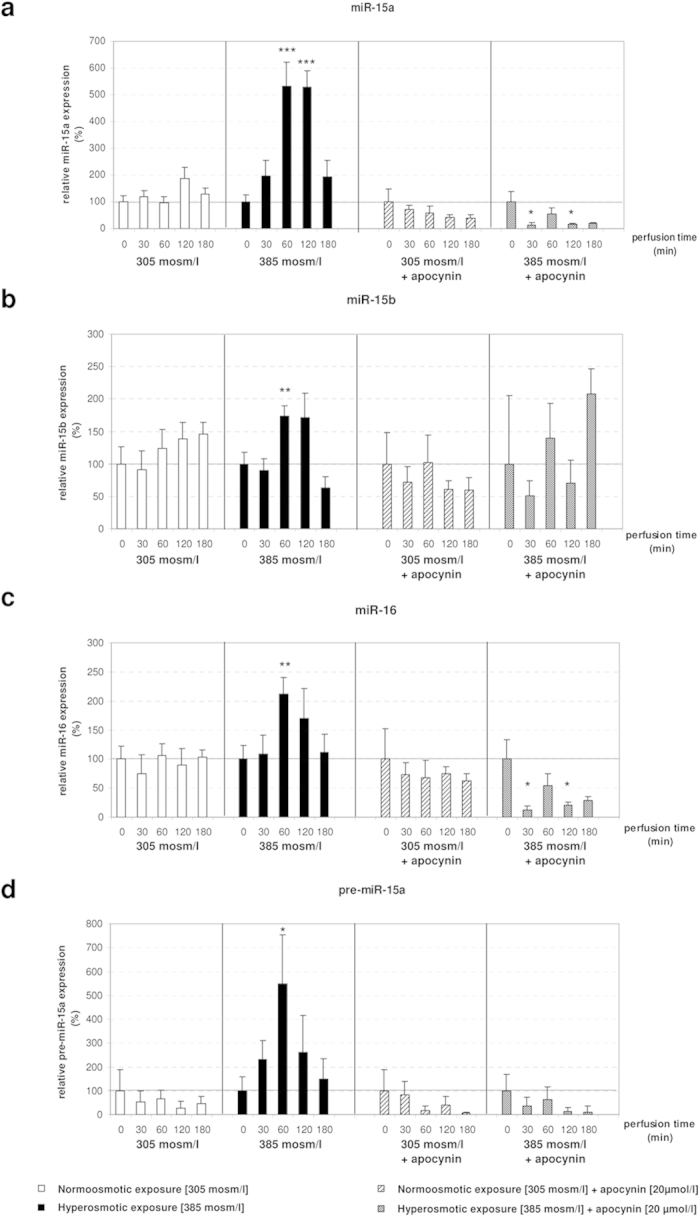
Upregulation of miR-15a/b and miR-16 by hyperosmolarity in perfused rat liver is blocked and downregulated by administration of apocynin. **(a)** After addition of apocynin (20 μmol/l) miR-15a is significantly downregulated at 30 and 120 minutes of hyperosmotic treatment. miR-15a levels are stable in the normoosmotic controls. **(b)** miR-15b is not changed after administration of apocynin in both hyperosmotically perfused livers and controls. **(c)** miR-16 is significantly downregulated after addition of apocynin at 30 and 120 minutes of hyperosmotic perfusion. **(d)** pre-miR-15a is significantly upregulated at 60 minutes of hyperosmotic stimulation, while this effect is inhibited by apocynin. qPCR runs were normalized according to the ΔΔCt method using RNU6 as reference gene. Statistical analysis was carried out by unpaired student’s t-test. Data are shown as average ± S.E.M. of 3 independent experiments. The values of unstimulated controls (T0) were set arbitrarily to 100.

It was previously shown that hyperosmotic exposure of rat hepatocytes triggers a proapoptotic state through the activation of the CD95 death receptor^4^,^5^. This process involves ROS formation via activation of NOX as upstream event^28^. In order to investigate whether ROS formation is involved in the regulation of miR-15a,-15b and -16, a new set of hyper- and normoosmotic perfusion experiments using the NOX inhibitor apocynin was performed. Following RNA isolation and quality control, the expression of miR-15 family members was measured by miQPCR. The analysis identified that apocynin was able to block the hyperosmotic activation of miR-15a, -15b and -16 (Fig. 3). Specifically, statistical analysis with unpaired student’s t-test indicates that miR-15a and miR-16 were significantly downregulated, while miR-15b expression was unchanged after addition of apocynin (Fig. 3). Altogether, these data indicate that NOX activation is required to modulate the activation of miR-15a, -15b and -16. Importantly, the finding that apocynin is able to suppress miRNA activation, strongly suggests that these miRNAs may belong to the redoximiRs, a novel miRNA grouping including all the redox sensitive miRNAs^29^.

**Figure 4:**
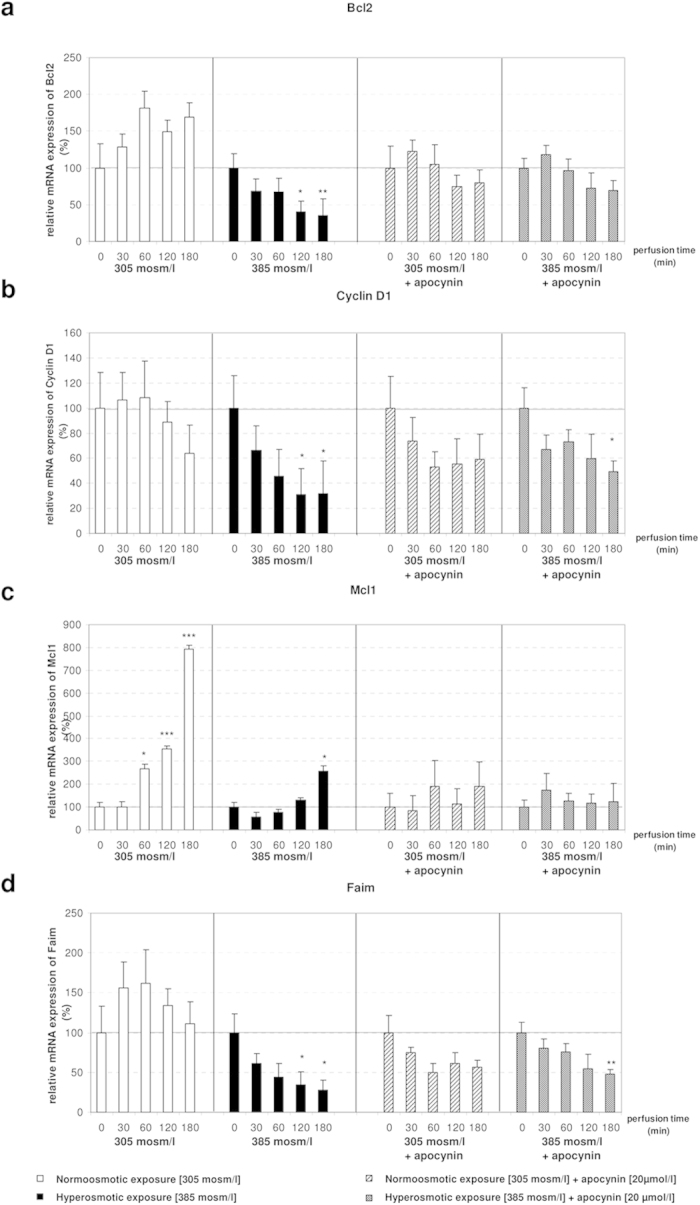
Bcl2 is not significantly changed at the mRNA level in hyperosmotically perfused rat livers after addition of apocynin. **(a)** Bcl2 is not significantly changed by hyperosmolarity after addition of apocynin. **(b)** Downregulation of Ccnd1 under hyperosmotic exposure is attenuated by administration of apocynin. **(c)** Mcl1 is not significantly changed in both normoosmotic and hyperosmotic conditions, when apocynin is added. **(d)** Faim is significantly downregulated after 180 minutes of hyperosmotic stimulation and apocynin, while it is not significantly changed after 120 minutes in the same experiment. qPCR runs were normalized according to the ΔΔCt method using β-Tubulin as reference gene. Statistical analysis was carried out by unpaired student’s t-test. Data are shown as average ± S.E.M. of 3 independent experiments. The values of unstimulated controls (T0) were set arbitrarily to 100.

In order to evaluate whether miRNA expression was regulated at the transcriptional level, expression of miR-15a precursor (pre-miR-15a) was measured by using miQPCR. Our analysis identified that pre-miR-15a was significantly upregulated under hyperosmotic conditions (Supplementary Figure 3a), while this effect was blocked by administration of apocynin (Fig. 3d). These data point to a transcriptional regulation of miR-15a expression. Correspondingly, the finding that apocynin was also able to largely prevent the hyperosmolarity-induced downregulation of Bcl2, Ccnd1, Mcl1 and Faim (Fig. 4) confirms the inverse correlation between the levels of these miRNAs and the expression of anti-apoptotic genes. These findings suggest that NOX activity is required to mediate both the upregulation of miR-15a, -15b and -16 and the downregulation of Bcl2, Ccnd1, Mcl1 and Faim under hyperosmotic conditions.

**Figure 5:**
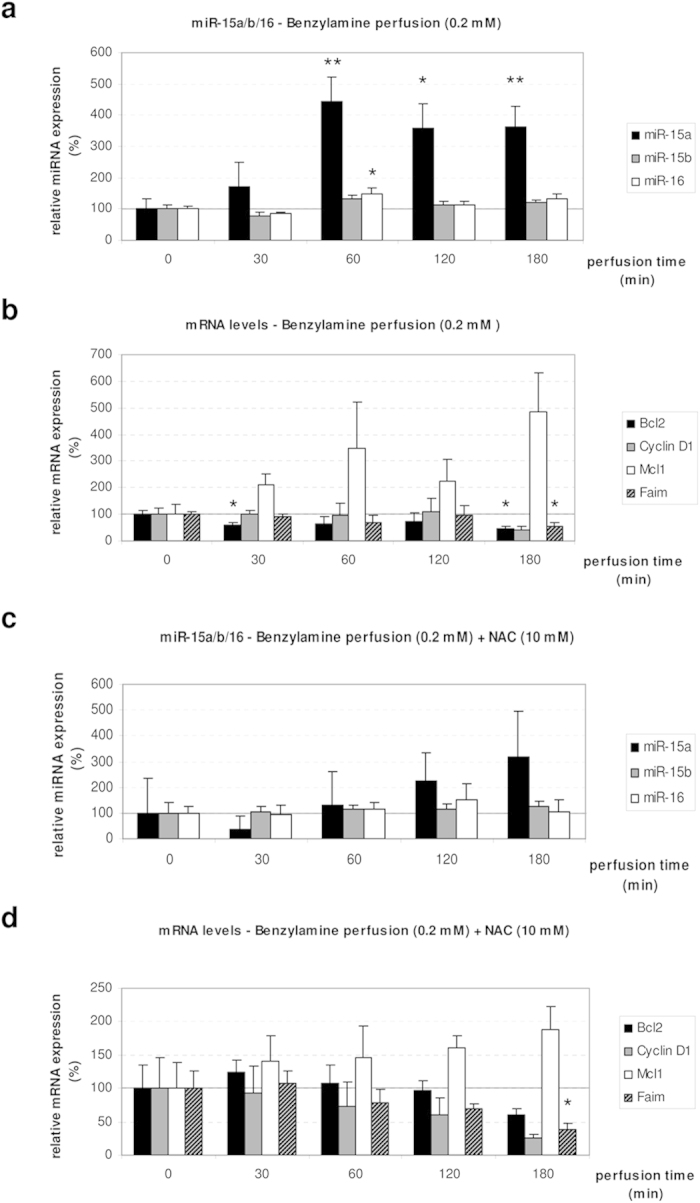
Benzylamine upregulates miR-15a/16 in a NAC-sensitive manner. Rat livers were perfused with benzylamine (0.2 mmol/l) and glucose (5 mmol/l). In the control, NAC was added with a concentration of 10 mmol/l. **(a)** miR-15a is significantly upregulated after 60 (p = 0.0031), 120 (p = 0.0184) and 180 minutes (p = 0.0062) of benzylamine perfusion. miR-16 is significantly upregulated after 60 minutes of benzylamine perfusion (p = 0.0245). **(b)** The target gene Bcl2 is significantly downregulated after 30 (p = 0.0421) and 180 minutes (p = 0.0131) of benzylamine perfusion, while Faim is significantly downregulated after 180 minutes of benzylamine perfusion (p = 0.022). **(c)** In presence of NAC the benzylamine-induced activation of miRNA was inhibited. **(d)** Bcl2, Cyclin D1 and Mcl1 are unchanged in presence of NAC, while Faim is significantly downregulated after 180 minutes of perfusion (p = 0.0468). qPCR runs were normalized according to the ΔΔCt method using β-tubulin as reference gene. Statistical analysis was carried out by unpaired student’s t-test. Data are shown as average ± S.E.M. of 3 independent experiments. The values of unstimulated controls (T0) were set arbitrarily to 100.

In order to substantiate that ROS is the driving factor mediating the observed miRNA downregulation, rat livers were perfused with benzylamine, which triggers as a substrate of monoamine oxidase the intracellular formation of H_2_O_2_ in liver and hepatocyte shrinkage^30^,^31^. Notably, the levels of miR-15a (p = 0.0031 after 60 minutes, p = 0.0184 after 120 minutes, p = 0.0062 after 180 minutes) and miR-16 (p = 0.0245 after 60 minutes) were significantly upregulated by this treatment (Fig. 5a), while Bcl2 (p = 0.0421 after 30 minutes, p = 0.0131 after 180 minutes) and Faim (p = 0.022 after 180 minutes) were significantly downregulated (Fig. 5b). On the other hand, there was a trend for Ccnd1 downregulation after 180 minutes of benzylamine perfusion (p = 0.0544), while Mcl1 expression was not affected by the treatment (Fig. 5b). In presence of the antioxidant N-acetylcysteine (NAC) the effects of benzylamine, on miRNA expression were no longer observed (5c–d). Taken together, these findings strengthen the view that ROS is required to mediate the observed response and that miR-15a, -15b and -16 are redoximiRs.

### ROS-mediated activation of JNK is required to activate the expression of miR-15a, miR-15b and miR-16

**Figure 6:**
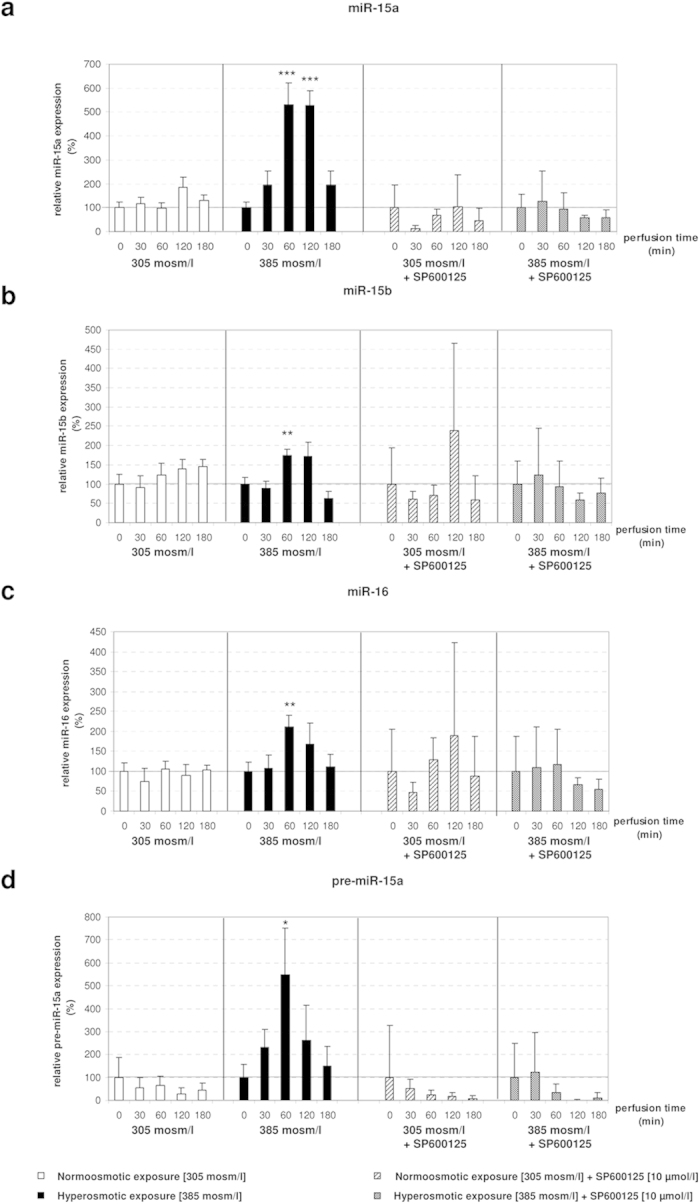
Upregulation of miR-15a/b and miR-16 by hyperosmolarity in perfused rat liver is blocked by administration of SP600125. **(a–d)** Addition of the JNK inhibitor SP600125 does not lead to significant changes of miRNAs under both hyperosmotic and normoosmotic conditions. qPCR runs were normalized according to the ΔΔCt method using RNU6 as reference gene. Statistical analysis was carried out by unpaired student’s t-test. Data are shown as average ± S.E.M. of 3 independent experiments. The values of unstimulated controls (T0) were set arbitrarily to 100.

**Figure 7:**
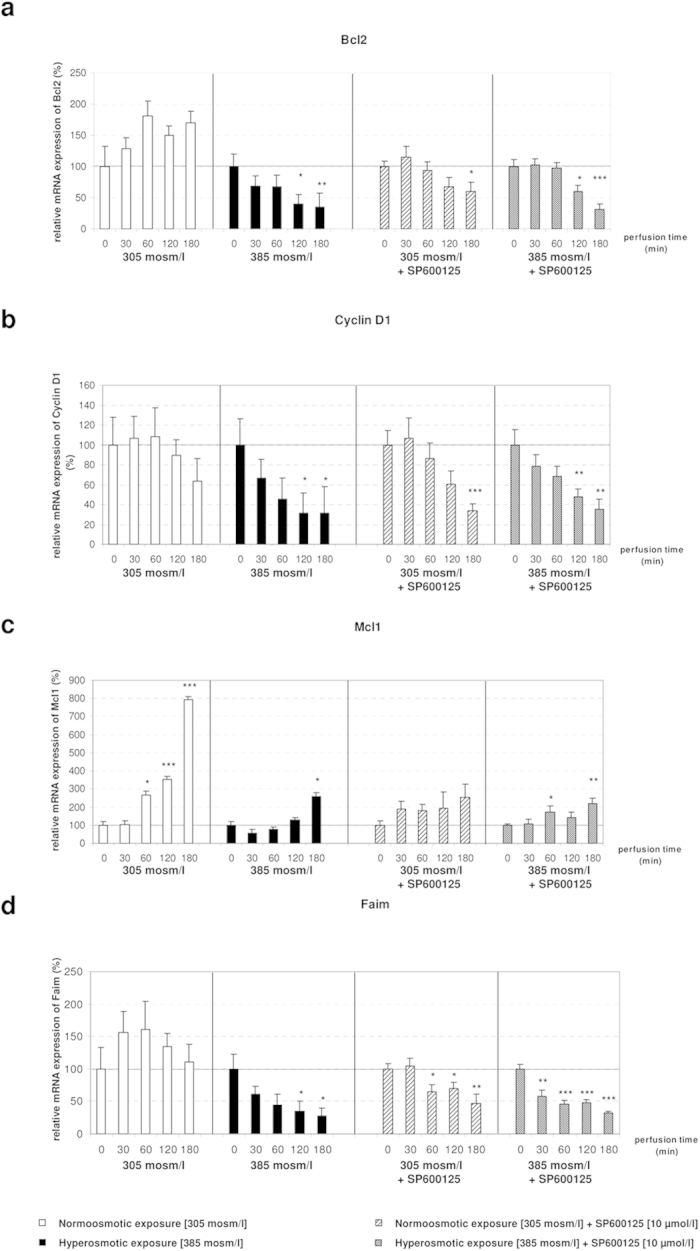
Effect of the JNK inhibitor SP600125 on the expression of Bcl2, Ccnd1, Mcl1 and Faim. **(a,b,d)** In presence of the JNK inhibitor SP600125 the effects of hyperosmolarity on the expression of Bcl2, Ccnd1 and Faim are largely abolished. **(c)** SP600125 inhibits Mcl1 mRNA expression in normoosmotic perfusions and in presence of SP600125 the expression-lowering effect of hyperosmolarity is largely abolished. qPCR runs were normalized according to the ΔΔCt method using β-Tubulin as reference gene. Statistical analysis was carried out by unpaired student’s t-test. Data are shown as average ± S.E.M. of 3 independent experiments. The values of unstimulated controls (T0) were set arbitrarily to 100.

NADPH oxidase-derived ROS controls the activity of several signaling pathways including JNKs, which is important for induction of CD95-mediated apoptosis^28^,^32^. In order to investigate whether JNK signaling is required to mediate the observed phenotype, SP600125, a potent JNK inhibitor^33^, was added to the perfusion medium 30 minutes before administration of SP600125-containing hyper- and normoosmotic solutions. We found that SP600125 largely counteracted the hyperosmotic activation of miR-15a, -15b, -16 and pre-miR-15a expression (Fig. 6). Next, we evaluated the effect of SP600125 on the expression levels of miR-15a, -15b and -16 target genes. Surprisingly, we found that Bcl2, Ccnd1 and Faim were still downregulated in response to hyperosmotic exposure in combination with SP600125, whereas Mcl1 was upregulated compared to T0 under the same conditions (Fig. 7). However, addition of SP600125 in the normoosmotic control also downregulated Bcl2, Ccnd1 and Faim and upregulated Mcl1 in the livers of control perfused animals. Based on these data, it is concluded that JNK inhibition by SP600125 prevents the hyperosmolarity-mediated downregulation of Bcl2, Ccnd1, Mcl1 and Faim. Overall, these data confirm that the observed regulation of miR-15a, -15b and -16 by hyperosmolarity is down-stream to NOX and requires JNK activity.

### Microarray analysis of hyperosmotically perfused liver identifies several osmoregulated genes

**Figure 8:**
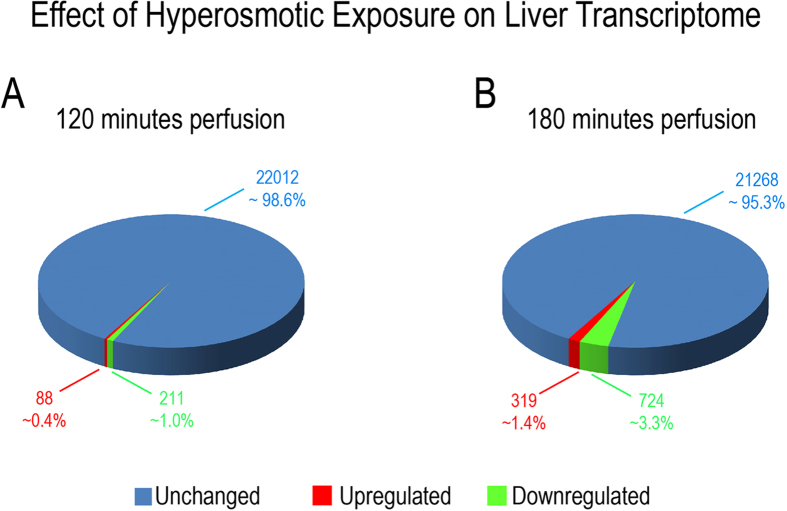
Overview on transcriptome changes in total RNA isolated from rat livers. Overview on transcriptome changes in total RNA isolated from rat livers perfused with normoosmotic (305 mosm/l) and hyperosmotic (385 mosm/l) medium for **(A)** 120 and **(B)** 180 minutes. RNAs isolated from 4 independent experiments for each time point were hybridized to Affymetrix arrays (rat Genechip v1.0) and data were analysed with AltAnalyze using a cut-off of 2-fold (significance level was set to p = 0.05; one-way ANOVA). After 120 minutes of hyperosmolarity 1.3% of the transcripts were significantly altered, while after 180 minutes of hyperosmolarity 4.7% of the transcripts were significantly altered. Upregulated genes are shown in red, downregulated genes are shown in green and unchanged genes are shown in blue.

**Figure 9:**
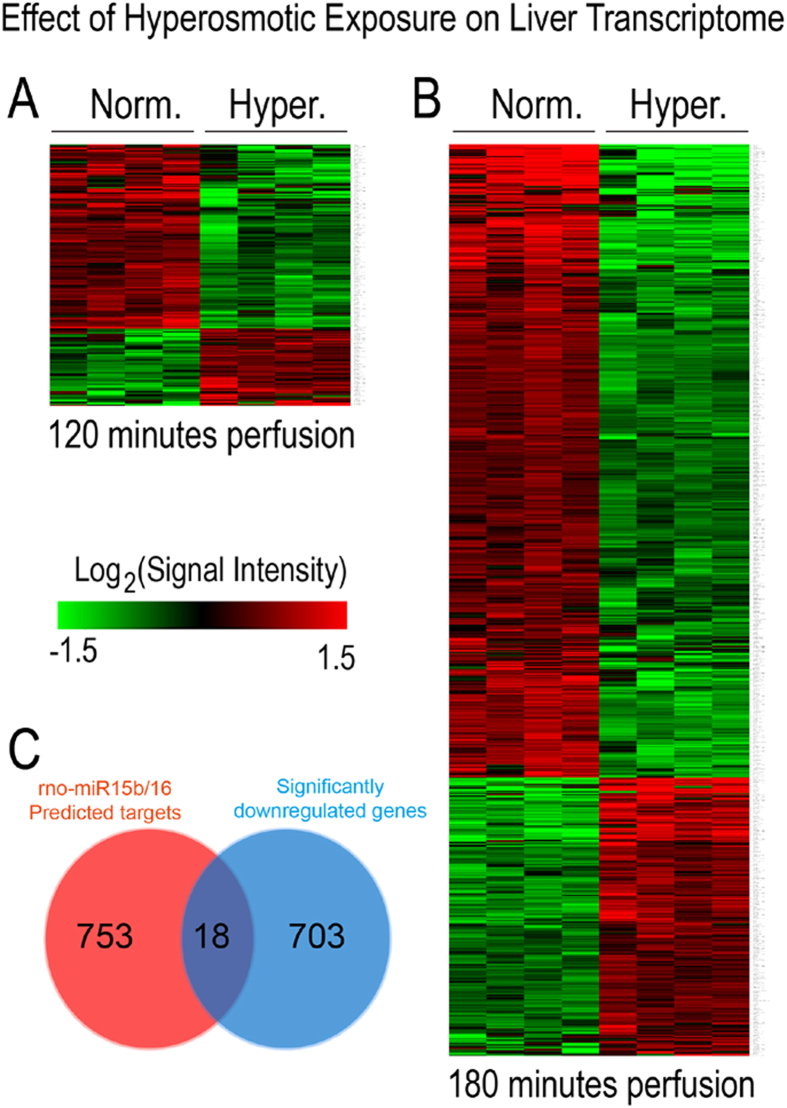
Heat map of genes with altered expression levels under hyperosmolarity. Hierarchical clustering representing the differential expression of significantly regulated genes in rat livers perfused with normoosmotic (305 mosm/l) and hyperosmotic (385 mosm/l) medium for 120 and 180 minutes. Hierarchical tree of gene clusters was generated by applying the Cousine Matrix. **(A)** 88 transcripts are upregulated, while 211 transcripts are downregulated under hyperosmotic exposure at 120 minutes. **(B)** 319 transcripts are upregulated, while 724 transcripts are downregulated under hyperosmotic exposure at 180 minutes. **(C)** Venn diagram presenting the number of genes and the overlap of miR-15a/b and miR-16 predicted target genes and the downregulated genes on the Affymetrix Chip. Target prediction analysis of the miR-15a/b and miR-16 was carried out by using the miRWalk algorithm.

**Figure 10:**
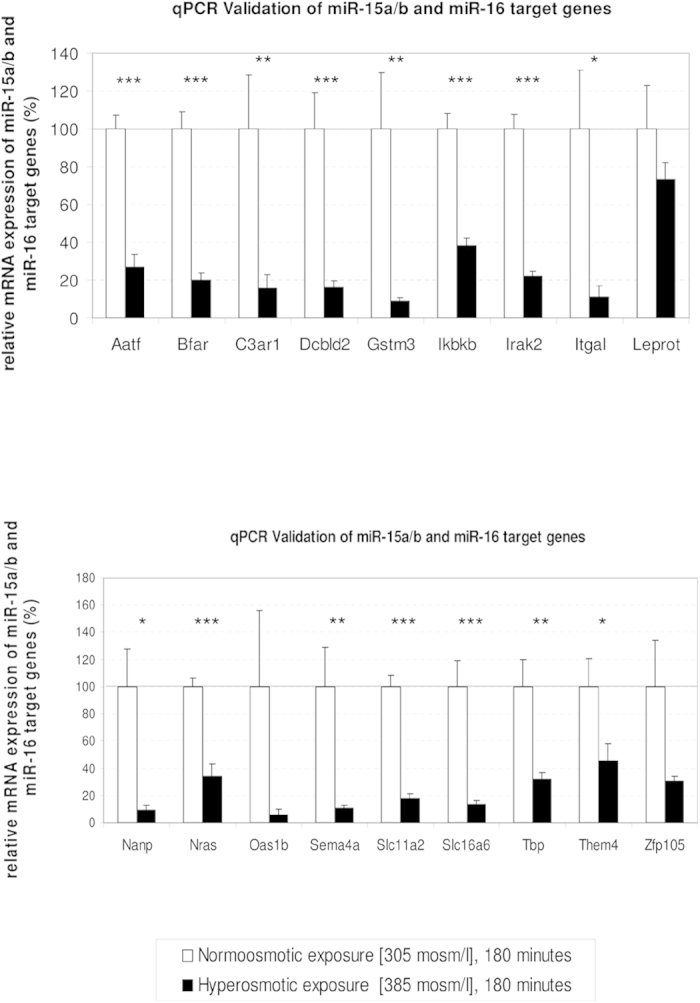
qPCR validation of potential target genes of the miR-15a/b and miR-16. qPCR validation of the 18 putative targets of miR-15a, miR-15b and miR-16 detected as significantly downregulated by Affymetrix arrays in hyperosmotically perfused livers. Downregulation of Aatf, Bfar, and Ikbkb may contribute to the promotion of a proapoptotic phenotype. Statistical analysis was carried out by unpaired student’s t-test. Data are shown as average ± S.E.M. of 4 independent experiments. qPCR runs were normalized according to the ΔΔCt method using β-Tubulin as reference gene. The values of unstimulated controls were set arbitrarily to 100.

In order to gain insight into the molecular mechanisms down-stream of ROS-mediated regulation of gene expression, genome-wide analysis of liver transcriptomes was carried out by using microarrays. Data analysis identified that application of hyperosmotic stress affected only a small fraction of transcripts. Specifically, we found that hyperosmotic treatment significantly affected 1.3% (299) and 4.7% (1043) of the genes present on the arrays within 120 minutes and 180 minutes, respectively (Fig. 8). Of the regulated transcripts, 88 (0.4%) and 211 (1%) were up- or downregulated, respectively after 120 minutes under hyperosmotic condition, whereas 319 (1.4%) and 724 (3.3%) transcripts were up- or downregulated after 180 minutes of hyperosmotic perfusion, respectively (Fig. 8 and Fig. 9A–B). Overall, the relatively small number of genes undergoing expression alterations upon the treatment suggests that hyperosmotic stress does not trigger cascades leading to catastrophic events. In order to identify the footprint of miR-15a, -15b and -16 on gene expression in a genome wide scale, putative miR-15b and -16 targets obtained from the miRWalk algorithm (771 predicted targets) were compared with the downregulated genes at 180 minutes after hyperosmotic exposure (721 genes). As shown in Fig. 9C, a total of 18 overlapping genes was identified (see also Table 1). Notably, three of the genes included in the list, namely the apoptosis antagonizing factor (Aatf), bifunctional apoptosis regulator (Bfar) and the inhibitor of kappa light polypeptide gene enhancer in B-cells, kinase beta (Ikbkb) are known for their anti-apoptotic activity^34^,^35^,^36^,^37^,^38^. Importantly, the significant downregulation of Aatf, Bfar, Ikbkb and of other 12 predicted targets could be validated by using qPCR (Fig. 10). For the remaining 3 predicted targets [Leptin receptor overlapping transcript (Leprot), 2-5 oligoadenylate synthetase 1B (Oas1b) and the zinc finger protein 105 (Zfp 105)], a tendency towards downregulation could be shown. Altogether, these data indicate that the upregulation of miR-15a, -15b and -16 might repress the expression of several anti-apoptotic genes potentially impairing cell survival.

### Transcription factors Foxo3a and Egr1 are potential candidates in the modulation of the miR-15 family

**Figure 11:**
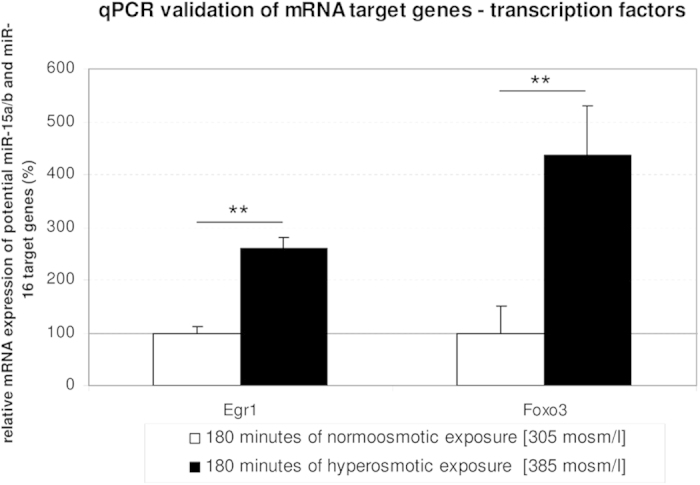
Transcription factors Egr1 and Foxo3 are osmoregulated genes. The transcription factors Egr1 and Foxo3 are significantly upregulated after 180 minutes of hyperosmotic stimulation. Statistical analysis was carried out by unpaired student’s t-test. Data are shown as average ± S.E.M. of 4 independent experiments. qPCR runs were normalized according to the ΔΔCt method using β-Tubulin as reference gene. The values of unstimulated controls were set arbitrarily to 100.

To gain an insight into the signaling pathways potentially regulated by hyperosmolarity in perfused rat liver, Gene Ontology (GO) enrichment was carried out for the list of up- and downregulated genes at 180 minutes (Supplementary Figure 4). Interestingly, GO Term enrichment analysis identified several highly enriched pathways, which are associated to ‘cell death’ (GO:0043067, p = 2.03 × 10^−9^; GO:0010941, p = 6.81 × 10^−9^ and GO:0012501,  = 1.83 × 10^−7^) and ‘apoptosis’ (GO:0042981, p = 1.13 × 10^−9^; GO:0043065, p = 1.3 × 10^−6^ and GO:0043066, p = 4.26 × 10^−6^; Supplementary Figure 4a). Whereas, among the upregulated genes the ‘regulation of transcription from RNA polymerase II promoter’ (GO:0006357, p = 5.08 × 10^−14^; Supplementary Figure 4b) was the pathway with the highest enrichment, suggesting that hyperosmotic treatment could potentially affect the expression of factors controlling transcription. Importantly, correlation among the 57 genes included in this GO term with literature mining suggests that the transcription factors Foxo3 and Egr1 are potential candidates for mediating the transcriptional regulation of miR-15a, -15b and -16^39^,^40^. Notably, Foxo3 was shown to upregulate miR-30d in renal carcinoma, thereby inducing cell cycle arrest and apoptosis^41^. Egr1 was shown to be upregulated by H_2_O_2_, and to play a crucial role in the transcriptional regulation of miR-20b in breast cancer^42^. Importantly, microarray data validation by qPCR confirmed the significant upregulation of both, Egr1 (2.6-folds; p = 0.008) and Foxo3 (4.4-folds; p = 0.003) after 180 minutes of hyperosmotic perfusion when compared to the normoosmotic control (Fig. 11). Further studies are required to substantiate the link between hyperosmotic Egr1/Foxo3 regulation and miR-15/16 expression.

## Discussion

As shown recently, hyperosmotic hepatocyte shrinkage triggers a ligand-independent CD95 death receptor activation, which involves as initial steps within seconds a chloride-driven acidification of endosomes with subsequent activation of acidic sphingomyelinase and ceramide formation, activation of protein kinase Cζ and activating phosphorylation of p47^phox^, a regulatory subunit of NADPH oxidase^4^,^5^,^28^,^43^. The accompanying ROS formation triggers JNK and Yes activation and downstream EGFR activation, EGFR/CD95 association followed by an EGFR-catalyzed CD95 tyrosine phosphorylation which initiates CD95 oligomerization, translocation of the CD95/EGFR complex to the plasma membrane and recruitment of the death-inducing signaling complex^5^,^44^,^45^,^46^. Whereas these processes occur on a short-term time scale, little is known about proapoptotic long-term effects of hyperosmotic cell shrinkage.

This study shows that miRNAs play a role in modulating cellular responses to hyperosmotic stress. Specifically, expression of miR-15a, miR-15b and miR-16 was found to be significantly upregulated in the livers of hyperosmotically perfused rats. Noticeably, miR-15a, -15b and -16 are tumor suppressor miRNAs, as reduction in their expression either caused by the deletion of the genomic locus containing the miR15a/16 cluster or epigenetic silencing is frequently detected in B-cell chronic lymphocytic leukemia (CLL). Moreover, these miRNAs, which are all members of the miR-15/107 family, serve key functions as they are known for repressing the expression of genes involved in cell division and metabolism^47^. Above all, miR-15a, -15b and -16 are thought to regulate apoptosis through the inhibition of anti-apoptotic genes including Bcl2 and Ccnd1^23^,^25^.

Our analysis shows that hyperosmotic perfusion of rat liver significantly downregulates the expression of anti-apoptotic genes including Bcl2, Ccnd1, Mcl1, Faim, Aatf, Bfar and Ikbkb at the mRNA level (Fig. 2 and Fig. 10). Notably, interaction between miR-15/16 and the 3′UTRs of both Bcl2 and Ccnd1 has been already validated in human, mouse and rat^23^,^24^,^25^. Interestingly, literature search indicates the anti-apoptotic gene Mcl1 as a miR-15a target^47^. However, this report could not be validated and, to the best of our knowledge, Mcl1 has been reported only as miR-15a predicted target in human^27^. Hence, to gain an insight into the correlation between the observed upregulation of miR-15a, -15b and -16 and the downregulation of anti-apoptotic genes, extensive bioinformatics analyses of data sets were carried out. Through this analysis, Faim, Aatf, Bfar and Ikbkb were identified as novel putative targets of miR-15a, -15b and -16 in the rat (Supplementary Figure 5). Importantly, Faim was shown to act as anti-apoptotic gene in B-cells^48^ and in the nervous system^49^. Moreover, Faim knock-out mice exhibit an increased sensitivity to Fas-triggered apoptosis, which results in a greater activation of caspase 8 and caspase 3 through decreased expression of c-FLIP^50^. The experimental model used for these experiments, namely the isolated perfused liver is an extremely powerful system. However, it has also limitations. For instance, the liver cannot be perfused indefinitely and it does not allow to evaluate the contribution of individual hepatic cell types to the observed response. In order to identify a complementary *in-vitro* system, primary rat hepatocytes were incubated with normo-, hyper- and hypoosmotic solution and the effect of the treatment on miRNA expression was evaluated by qPCR (Supplementary Figure 6). However, as no significant changes were identified, it was concluded that cell-cell and/or cell-matrix interactions within the intact liver might be a prerequisite for the regulation of osmotically sensitive miRNAs. In this regard, it should be noted that osmosensing and osmosignaling in hepatocytes requires integrin-matrix interactions^3^. However, the possibility is not excluded that liver cell types other than parenchymal cells, such as hepatic stellate cells, Kupffer cells or *liver sinusoidal* endothelial *cells* might contribute to the effects seen in anisoosmotically perfused liver.

The finding that NOX and JNK inhibitors could largely prevent hyperosmolarity-induced upregulation of miR-15a, -15b and -16 expression (Figs 3 and 6) indicates that these miRNAs, either directly or indirectly, respond to ROS. Moreover, the observation that the apocynin-mediated inhibition of NOX under hyperosmotic conditions prevented also the downregulation of Bcl2, Ccnd1, Mcl1 and Faim expression (Fig. 4), indicates that ROS activity is required to mediate both the observed activation of miRNA and inhibition of mRNA expression. In addition, endogenous ROS-generation at monoamine oxidase by benzylamine had similar effects on miRNA expression and was inhibited by NAC (Fig. 5). Overall, these findings support the hypothesis that miR-15a, -15b and -16 are redoximiRs. Based on this, we propose that the response of these miRNAs to osmotic stress could identify a novel mechanism in the regulation of redox signaling.

miRNA expression can be regulated at both transcriptional or post-transcriptional levels via the modulation of miRNA biogenesis or by affecting miRNA turn-over. In order to evaluate whether miRNA expression is regulated at transcriptional or post-transcriptional level, miQPCR was used to measure the level of miR-15a and -16 precursors (Supplementary Figure 3). Our data indicate that different types of regulation might be active, as a significant upregulation was observed for the pre-miR-15a (Supplementary Figure 3a) under hyperosmotic stimulation, whereas levels of pre-miR-16 were unchanged (Supplementary Figure 3b). The finding that both, apocynin and SP600125 were able to inhibit hyperosmolarity-induced upregulation of pre-miR-15a (Fig. 3d and Fig. 6d), strongly supports the hypothesis that expression of miR-15a is regulated by ROS at transcriptional level. Further work is required to fully characterize the ROS-effects on the biogenesis of miR-15a, -15b and -16.

In order to gain more insight into the effects of osmotic cell regulation, genome wide analysis of RNA transcription was carried out. Enrichment analysis of significantly regulated genes indicates that hyperosmotic stimulation affects several biological processes (Supplementary Figure 4). Importantly, we found that among the genes downregulated within 180 minutes of hyperosmotic perfusion, 18 genes were predicted target genes of miR-15b and miR-16 (Table 1 and Fig. 10). Interestingly, it was identified that three of the genes, namely Aatf, Bfar and Ikbkb are anti-apoptotic genes. Aatf ameliorates mitochondrial dysfunction by reducing oxidative damage^34^. Bfar is a multidomain protein that interacts with members of the extrinsic and intrinsic apoptosis pathways^35^ and inhibits JNK signaling^36^. JNK signaling might thus be activated by downregulation of Bfar. Furthermore, Bfar has been reported to be downregulated in response to oxidative stress induced by H_2_O_2_ and peroxynitrite and under hypoxic conditions^35^. Embryonic development of IKK-β knock out mice is affected by massive liver degeneration^51^ as NF-κB protects the liver against TNFα-induced apoptosis and mice lacking the catalytic subunit of NF-κB IKK-β were reported to die because of increased hepatocyte apoptosis^37^. Interestingly, downregulation of IKK-β is also critical for triggering JNK-dependent cell apoptosis in HepG2 cells under arsenite exposure^52^. Taken together, hyperosmotic stress in perfused rat liver downregulates several anti-apoptotic genes, potentially promoting a proapoptotic phenotype in addition to the previously described short-term activation of the CD95 death receptor system^4^,^5^,^46^.

**Figure 12:**
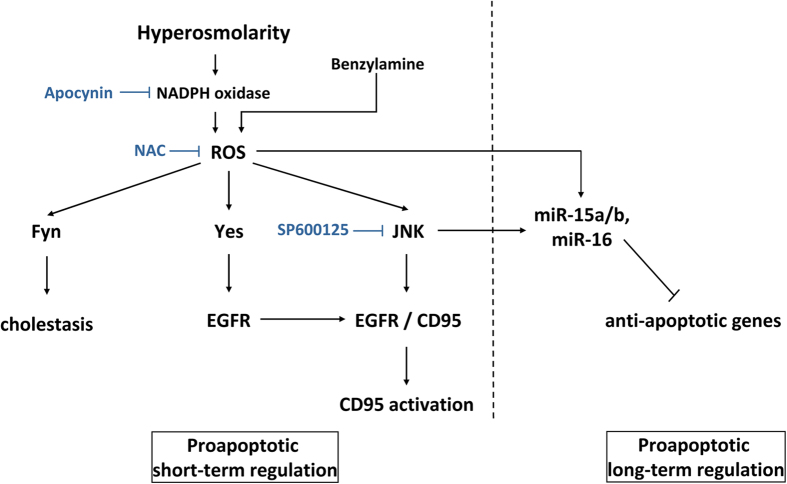
Schematic representation of short and long term hyperosmotic signaling towards apoptosis. Hyperosmotic hepatocyte shrinkage induces Nox-driven generation of ROS through a signaling pathway involving endosomal acidification, acidic sphingomyelinase activation, ceramide-dependent PKCζ activation and serine phosphorylation of p47^phox^, the regulatory subunit of Nox^4^,^5^,^28^,^43^. ROS formation activates JNK^4^ which may directly or indirectly regulate transcription factors being required for the regulation of apoptotic genes and the biogenesis of the miR-15/107 family. Activated miRNAs in turn repress anti-apoptotic genes on a posttranscriptional level. In contrast to the short term signaling pathway towards CD95 activation (involving EGFR activation, EGFR/CD95 association, CD95 tyrosine phosphorylation and oligomerization, translocation of the CD95/EGFR complex to the plasma membrane and recruitment of the death-inducing signaling complex^4^,^5^,^6^,^28^,^44^,^45^,^46^), miR-15a/b/-16 upregulation is a long-term proapoptotic regulatory mechanism. Furthermore, ROS is generated by benzylamine through the monoamine oxidase reaction^30^ and may inhibit the expression of anti-apoptotic genes.

Up to now, regulation of miRNA transcription is still poorly understood. Reason for this is that with the exclusion of intronic miRNAs, which are supposedly co-transcribed from the promoter of the host gene, there has been very little progress in the field of miRNA promoters. With the purpose to identify putative transcription factors that could mediate regulation of miRNA transcription, GO term enrichment was applied to the upregulated genes after 180 minutes of hyperosmotic stress (Supplementary Figure 4b). Importantly, our analysis suggests that the transcription factors Foxo3 and Egr1 could be possible drivers of the hyperosmotic-specific activation of miR-15/16 expression. Specifically, both Foxo3 and Egr1 have been described as stress-responsive TFs, mediating the activation of stress responsive genes^39^,^40^. Figure 12 summarizes our current view on the role of miRNA in hyperosmotic signaling in the liver.

## Methods

### Liver perfusion

One of the established experimental models to investigate osmotic stress is the perfusion of isolated rat livers^53^. Importantly, this experimental system enables for both the application of biochemical assays and the monitoring of the metabolic changes associated to various diseases. Livers from male Wistar rats (fed *ad libitum*) were perfused after an initial 20 min preperfusion with normoosmotic medium with either normo-, hyper- or hypoosmotic fluid (305, 385 and 225 mosm/l respectively), for 0, 30, 60, 120 and 180 minutes as previously described^54^. The perfusion medium was bicarbonate-buffered Krebs-Henseleit-medium equilibrated with O_2_/CO_2_ (95/5, *v/v*) and supplemented with lactate (2.1 mmol/l) and pyruvate (0.3 mmol/l) in a non-recirculating perfusion system at 37 °C. Osmolarity adjustment was achieved by corresponding changes in NaCl concentration. At the end of the perfusion experiment, liver tissue was cut in pieces and flash frozen in liquid nitrogen and stored at −80 °C until use. This study and all experimental protocols were approved and the methods were carried out in accordance with the guidelines of the Institutional Animal Care and Use Committee of the University Hospital Düsseldorf.

### RNA isolation and quality control

Liver fragments were removed from −80 °C and then homogenized using mortar and pestle on dry ice and lysed with QIAzol Lysis Reagent (Qiagen, Hilden, Germany). miRNAs were isolated by use of the RNeasy Mini Kit (Qiagen) by following the instructions provided with the kit. RNA concentration was measured with the Nanodrop 1000 (Thermo Scientific, Waltham, USA) and RNA integrity was assessed by both agarose gel electrophoresis or by the Bioanalyzer (Agilent, Santa Clara, USA).

### cDNA synthesis for microRNA and mRNA and analysis by qPCR

cDNA synthesis and primer design for the analysis of miRNA expression profiling by miQPCR was carried out as previously described^55^. Specifically, miQPCR is a novel approach to reverse transcribe universally all the miRNAs contained in the RNA sample. Importantly, cDNA synthesis according to the miQPCR method requires 10 ng of total RNA and produces enough material for running up to 100 individual qPCRs, equivalent to 100 pg cDNA/qPCR assay. qPCR assays were performed on a TProfessional Thermocycler (Biometra, Analytik Jena) with SensiMix SYBR Green No-ROX (Bioline, London, UK). For miRNA amplification the following program was used: 95 °C for 10 minutes (1 cycle), 95 °C for 15 seconds and 60 °C for 35 seconds (50 cycles), followed by melting curve. Primer efficiency was assessed by analysis of standard curves with three times 10-fold dilution series. qPCR was carried out in 5 independent experiments for miR-15a/b and miR-16 in hyper-, hypo- and normoosmotically treated samples. cDNA for mRNA analysis was carried out as previously described^55^. Briefly, 200 ng of total RNA were reverse transcribed by random priming (Thermo Scientific, Catalogue no. SO142) and PrimeScript reverse transcriptase (Takara, Catalogue no. 2680A) following vendor instructions and 5 μl of diluted cDNA (equivalent to 10 ng of RNA) were used in qPCR assays. For mRNA amplification the following protocol was used: 95 °C for 10 minutes (1 cycle), 95 °C for 15 seconds and 60 °C for 1 minute (40 cycles), followed by melting curve. For both miRNA and mRNA analysis primer efficiency was assessed by standard curves. Primers used in miRNA quantification and qPCR validation of targets genes are listed in Supplementary Tables 1 and 2. qPCR data were analysed by qBase software v.1.3.5^56^. Statistical analysis was carried out by unpaired student’s t-test. Significance level was set to p = 0.05.

### Western Blot analysis and Immunoprecipitation

#### Material

The Bcl2 recognizing antibody (#ab7973) was purchased from abcam (Cambridge, UK) and glyceraldehyde 3-phosphate dehydrogenase (GAPDH; #MAB374) from Merck-Millipore (Darmstadt, Germany). Antibodies recognizing CD95 (#sc-715; IP) were from Santa Cruz Biotechnology (Heidelberg, Germany), phospho-tyrosine (#05-321) from Merck-Millipore (Darmstadt, Germany), CD95 (#MA1-7622; WB) from Life Technologies GmbH (Darmstadt, Germany), cleaved caspase 3 (#9664), γ-tubulin (#T5326) from Sigma Aldrich (Munich, Germany).

#### Immunoblot analysis

Liver samples were immediately lysed at 4 °C by using a lysis buffer containing 20 mmol/l Tris-HCl (pH 7.4), 140 mmol/l NaCl, 10 mmol/l NaF, 10 mmol/l sodium pyrophosphate, 1% (*v/v*) Triton X-100, 1 mmol/l EDTA, 1 mmol/l EGTA, 1 mmol/l sodium vanadate, 20 mmol/l β-glycerophosphate, and protease inhibitor cocktail. The lysates were kept on ice for 10 minutes, centrifuged at 8000 rpm for 8 minutes at 4 °C, and aliquots of the supernatant were taken for protein determination using the Bio-Rad protein assay (Bio-Rad Laboratories, Munich, Germany). Equal amounts of protein were subjected to sodium dodecyl sulfate/polyacrylamide gel electrophoresis, and transferred onto nitrocellulose membranes using a semidry transfer apparatus (GE Healthcare, Freiburg, Germany). Membranes were blocked for 30 minutes in 5% (*w/v*) BSA containing 20 mmol/l Tris (pH 7.5), 150 mmol/l NaCl, and 0.1% Tween 20 (TBS-T) and exposed to primary antibodies overnight at 4 °C. After washing with TBS-T and incubation at room temperature for 2 hours with horseradish peroxidase-coupled anti-mouse or anti-rabbit IgG antibody, the immunoblots were washed extensively and bands were visualized using the FluorChem E detection instrument from ProteinSimple (Santa Clara, CA). Semi-quantitative evaluation was carried out by densitometry using the Alpha View image acquisition and analysis software from ProteinSimple. Change in protein expression is given as the ratio of detected Bcl2/GAPDH.

#### Immunoprecipitation

Liver samples were lysed in lysis buffer containing 136 mmol/l NaCl, 20 mmol/l Tris HCl, 10% (*v/v*) glycerol, 2 mmol/l EDTA, 50 mmol/l β-glycerophosphate, 20 mmol/l sodium pyrophosphate, 0.2 mmol/l Pefablock, 5 mg/l aprotinin, 5 mg/l leupeptin, 4 mmol/l benzamidine, 1 mmol/l sodium vanadate, supplemented with 1% (*v/v*) Triton X 100. The protein amount was determined as described above. Samples containing equal protein amounts were incubated for 2 h at 4 °C with an anti-CD95 antibody to immunoprecipitate CD95. Then protein A-/G-agarose (Santa Cruz Biotechnology, Heidelberg, Germany) was added and incubated at 4 °C overnight. Immunoprecipitates (IPs) were washed thrice with lysis buffer supplemented with 0.1% (*v/v*) Triton X-100 and then transferred to Western blot analysis as described above. The anti-phospho-tyrosine antibody was used to detect activating phosphorylation of CD95 in the respective IPs.

### Detection of ROS

Hepatocytes were seeded on collagen-coated 6-cm culture plates (BD Falcon, Heidelberg, Germany) and cultured for 24 h. Cells were incubated with PBS containing 5 μmol/l CM-H_2_DCFDA (Life Technologies), for 30 min at 37 °C in a humidified atmosphere of 5% CO_2_ and 95% air. To detect ROS generation, CM-H_2_DCFDA-loaded cells were washed and thereafter exposed to hyperosmotic medium (405 mosm/l) for 15 minutes. Thereafter cells were washed briefly using ice-cold PBS, and cells were lysed in 0.1 % Triton X-100 *(v/v)* dissolved in aqua bidest. Lysates were centrifuged immediately (10 000 × *g*, 4 °C, 1 min), and fluorescence of the supernatant was measured at 515–565 nm using a luminescence spectrometer LS-5B (PerkinElmer Life Sciences, Rodgau, Germany) at a 485 nm excitation wavelength. Fluorescence intensity of controls was set to 1 and fluorescence found under the respective treatment is given relative to it.

### Target prediction analysis

Target prediction analysis was carried out and target genes were analysed according to their potential binding sites for miRNAs in their 3′UTR. Putative miRNA targets were identified by use of the miRBase database and the gene database EnsEMBL as well as MicroCosm Targets (Version 5) and the CLC Genomics Workbench. miRNAs of interest were loaded onto MicroCosm and their potential targets were analysed by comparison of miRNA sequences with the corresponding seed sequences within the 3′UTR of target mRNAs. Multiple target genes were found and miRNAs, which are associated to liver pathology, were analysed.

### Bioinformatics

miR-15a, miR-15b and miR-16 are conserved between human, mouse and rat genome. Moreover, the seed sequences are identical meaning that these miRNAs belong to the same family (24; see also Supplementary Figure 5a). Bioinformatics analyses further revealed that the seed sequences of the miR-15 family are around 2,2 kb downstream of the 3′UTR of Bcl2, potentially targeting this miRNA which leads to inhibition.

### Online databases

miRNA sequences were acquired from www.mirbase.org (Version 21, release June 2014). Melting temperatures for miRNA primers were calculated by use of the online tool Tm calculator at http://www6.appliedbiosystems.com/support/techtools/calc/index.cfm. Primers for mRNA amplification were assessed by http://eu.idtdna.com/calc/ analyzer with respect to their secondary structure, possible self-dimerization and primer duplexes. MicroCosm Targets (Version 5) at http://www.ebi.ac.uk/enright-srv/microcosm was used for target prediction and from the Ensembl Genes database 78 at http://www.ensembl.org/biomart/martview/ genomic DNA sequences were obtained as well as the 3′UTR. miRWalk at http://www.umm.uni-heidelberg.de/apps/zmf/mirwalk/ was also utilized for target prediction analysis. Venn diagrams and intersection between gene lists were calculated with the freely available on-line tool Venny (http://bioinfogp.cnb.csic.es/tools/venny).

### Affymetrix microarray hybridization, data analysis and GO term enrichment

To identify at a genome wide level the effect of hyperosmotic perfusion on the liver transcriptome, hepatic RNAs isolated at time 0, 120 and 180 minutes (4 independent experiments) were hybridized to Affymetrix GeneChip rat gene 1.0 ST arrays. cDNA synthesis and labeling were performed according to the Affymetrix protocol. Hybridization and washing were carried out in a SciGene 777 Microarray Oven (50 rpm, 45 °C for 16 hours) and in an Affymetrix GeneChip 450 Washer respectively. Microarrays scanning and acquisition of signal intensities were performed on the Affymetrix GeneChip Scanner GCS3000 System. Microarray data analysis of the Affymetrix Rat Gene Chip 1.0 ST were performed by using the AltAnalyze software^57^ by using default setting. Default filter criteria were at least 2-fold changes on the linear scale and a p-value <0.05. Gene ontology (GO) term enrichment of biological process was performed by using GOrilla^58^. Specifically, significantly up- or downregulated genes at 180 minutes after hyperosmotic perfusion were compared to a background list including all the rat genes included in the Affymetrix GeneChip rat gene 1.0 ST as identified by EnsEMBL-Biomart (http://www.ensembl.org/biomart).

### Analysis of miRNAs in cell culture

Hepatocytes were isolated from livers of male Wistar rats by collagenase perfusion (as described in^5^), plated on collagen-coated 6-well culture plates (Greiner Bio One, Frickenhausen, diameter 35 mm) with aliquots of 0.67 × 10^6^ cells per well (total of 4 × 10^6^ hepatocytes per 6-well-plate) and exposed to either hyper-, hypo- or normoosmotic Williams E medium (405 mosm/l, 205 mosm/l and 305 mosm/l, respectively; supplemented with 5% FCS, 1% penicillin/streptomycin, 1% glutamine, 100 pM insulin and 100 nM dexamethasone). Osmolarity adjustment was achieved by corresponding changes in NaCl concentration. The viability of the hepatocytes was >95% as assessed by trypan blue exclusion. After 24 hours of incubation at 37 °C in a 5% CO_2_ atmosphere (relative humidity of 95%), hepatocytes were washed with PBS and harvested. Subsequently, RNA was isolated by the same method as described above (miRNeasy Mini Kit, Qiagen).

### Inhibitor studies

The inhibitors were added to the perfusion fluid, starting with a preperfusion 30 minutes before administration of hyperosmotic medium (385 mosm/l KHB). The NAPDH oxidase inhibitor apocynin was added at a concentration of 20 μmol/l to the perfusion medium (n = 3). The JNK inhibitor SP600125 was added to the perfusion medium with a concentration of 10 μmol/l (n = 3). As for control, the inhibitors were also added in normoosmotic control experiments. miRNAs were subsequently isolated from liver tissue with the miRNeasy Kit (Qiagen) as described above. Benzylamine was administered to a normoosmotic (305 mosm/l) and glucose (5 mmol/l)-containing medium at a concentration of 0.2 mmol/l. NAC was added in these experiments with a concentration of 10 mmol/l 30 minutes before administration of benzylamine.

## Additional Information

**How to cite this article**: Santosa, D. *et al*. Hyperosmotic stress activates the expression of members of the miR-15/107 family and induces downregulation of anti-apoptotic genes in rat liver. *Sci. Rep*. **5**, 12292; doi: 10.1038/srep12292 (2015).

## Supplementary Material

Supplementary Information

## Figures and Tables

**Table 1:** Description of the miR-15b and miR-16 putative targets downregulated under hyperosmotic conditions.

Gene Name	Description	Seed Length	Seed Sequence	Binding Start	Binding End	mRNA Region
Aatf	Apoptosis antagonizing transcription factor	7	AGCAGCA	1751	1745	3 UTR
Bfar	Bifunctional apoptosis regulator	8	AGCAGCAC	1606	1599	3 UTR
C3ar1	Complement component 3a receptor 1	7	UAGCAGC	1924	1918	3 UTR
Dcbld2	Discoidin, CUB and LCCL domain containing 2	8	AGCAGCAC	2334	2327	3 UTR
Gstm3	Glutathione S-transferase mu 3	7	AGCAGCA	1160	1154	3 UTR
Ikbkb	Inhibitor of kappa light polypeptide: enhancer in B-cell	9	UAGCAGCAC	2584	2576	3 UTR
Irak2	Interleukin-1 receptor-associated kinase 2	8	UAGCAGCA	2628	2621	3 UTR
Itgal	Integrin, alpha L	7	UAGCAGC	3960	3954	3 UTR
Leprot	Leptin receptor overlapping transcript	8	UAGCAGCA	1646	1639	3 UTR
Nanp	N-acetylneuraminic acid phosphatase	7	AGCAGCA	915	909	3 UTR
Nras	Neuroblastoma RAS viral oncogene homolog	7	AGCAGCA	771	765	3 UTR
Oas1b	2′-5′-oligoadenylate synthetase 1	8	UAGCAGCA	1776	1769	3 UTR
Sema4a	Sema domain, immunoglobulin domain	7	AGCAGCA	2781	2775	3 UTR
Slc11a2	Solute carrier family 11, member 2	8	UAGCAGCA	1998	1991	3 UTR
Slc16a6	Solute carrier family 16 member 6	7	AGCAGCA	1939	1933	3 UTR
Tbp	TATA box binding protein	9	UAGCAGCAC	1440	1432	3 UTR
Them4	Thioesterase superfamily member 4	8	AGCAGCAC	1342	1335	3 UTR
Zfp105	Zinc finger protein 105	8	UAGCAGCA	1861	1854	3 UTR

Analysis of both bioinformatics (miRWalk) and gene chip data led to 18 potential target genes of the miR-15a, miR-15b and miR-16.

Predicted binding sites within the 3′UTR of the potential target genes are listed. Among them, several genes are involved in apoptotic processes, such as Aatf, Bfar or Ikbkb.
