# Supplementary material for: Hyperosmotic stress activates the expression of members of the miR-15/107 family and induces downregulation of anti-apoptotic genes in rat liver
Source: Sci Rep. 2015 Jul 21;5:12292. doi: 10.1038/srep12292 (PMC4508667; doi:10.1038/srep12292)
Supplement: Supplementary Information [file srep12292-s1.pdf]

Supplementary Information

Hyperosmotic stress activates the expression of members of the miR-15/107 family and induces downregulation of anti-apoptotic genes in rat liver

David Santosa[1,+], Mirco Castoldi[1,+,], Martha Paluschinski[1], Annika Sommerfeld[1] and Dieter Häussinger[1,*]*

[1]Department of Gastroenterology, Hepatology and Infectious Diseases, Heinrich-Heine-University, Moorenstrasse 5, 40225 Düsseldorf, Germany.

[+]these authors contributed equally to this work

***for correspondence**

Dieter Häussinger, haeussin@uni-duesseldorf.de

Mirco Castoldi, mirco.castoldi@uni-duesseldorf.de

Department of Gastroenterology, Hepatology and Infectious Diseases

Heinrich-Heine-University Düsseldorf

Moorenstrasse 5

D-40225 Düsseldorf/Germany

Fax (49) 211 8118838

Key words

Liver perfusion, osmotic stress, oxidative stress, ROS, microRNA, redoximiR, apoptosis

Supplementary Figure 1

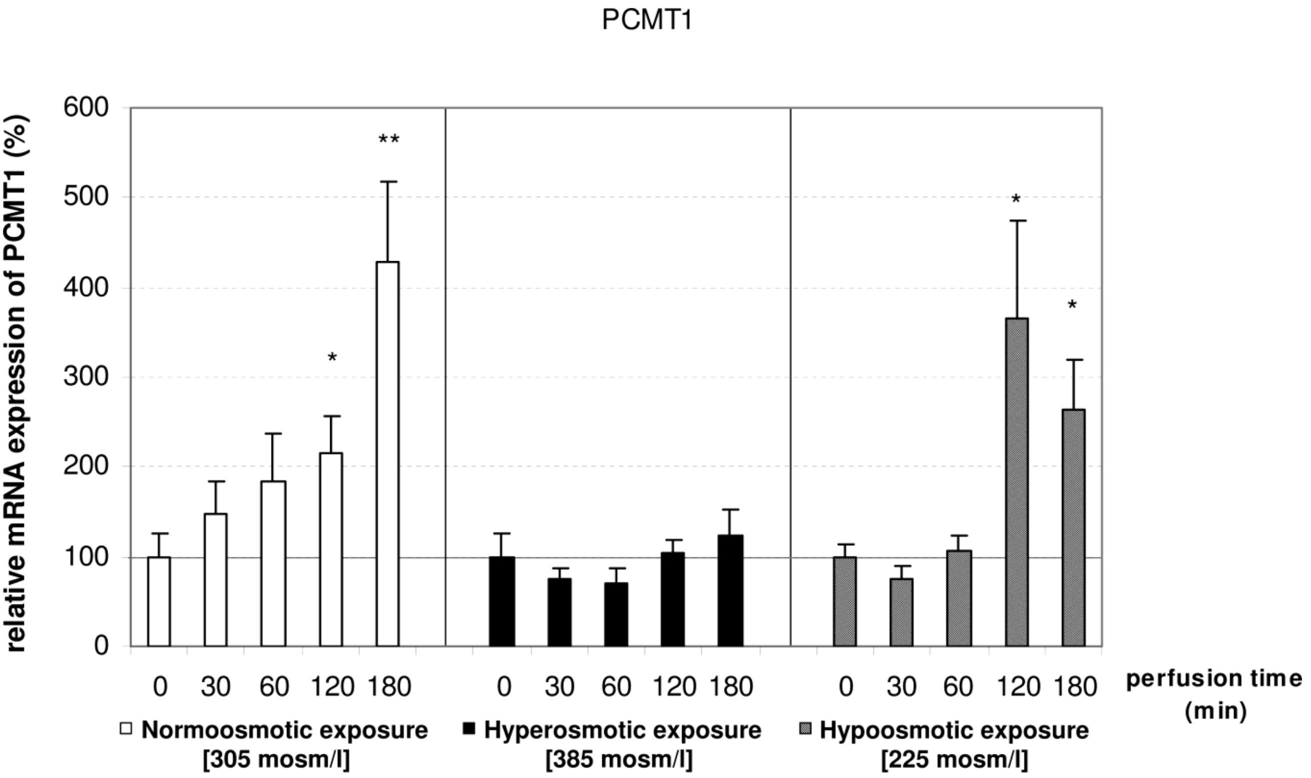

Supplementary Figure 2

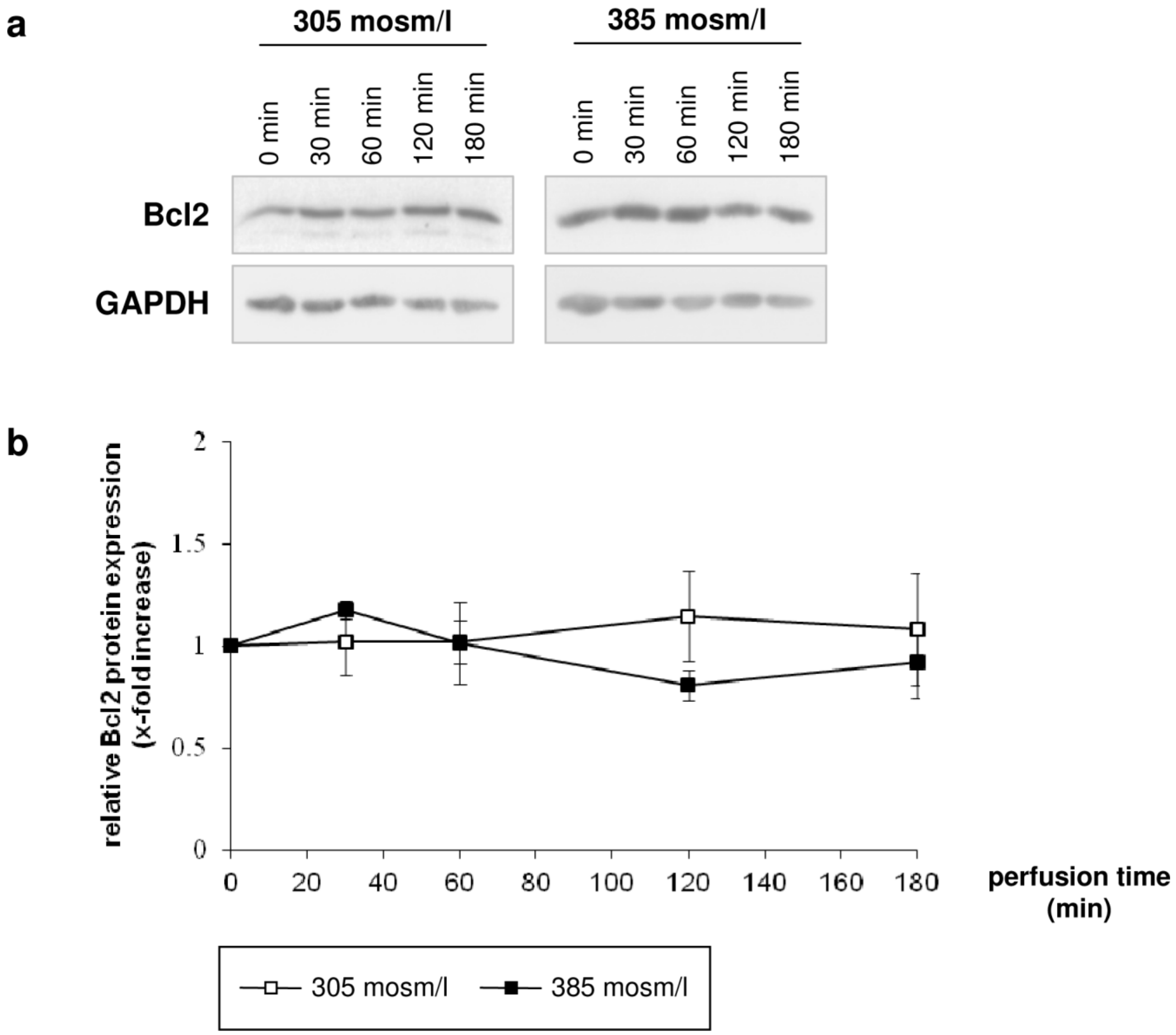

Supplementary Figure 3

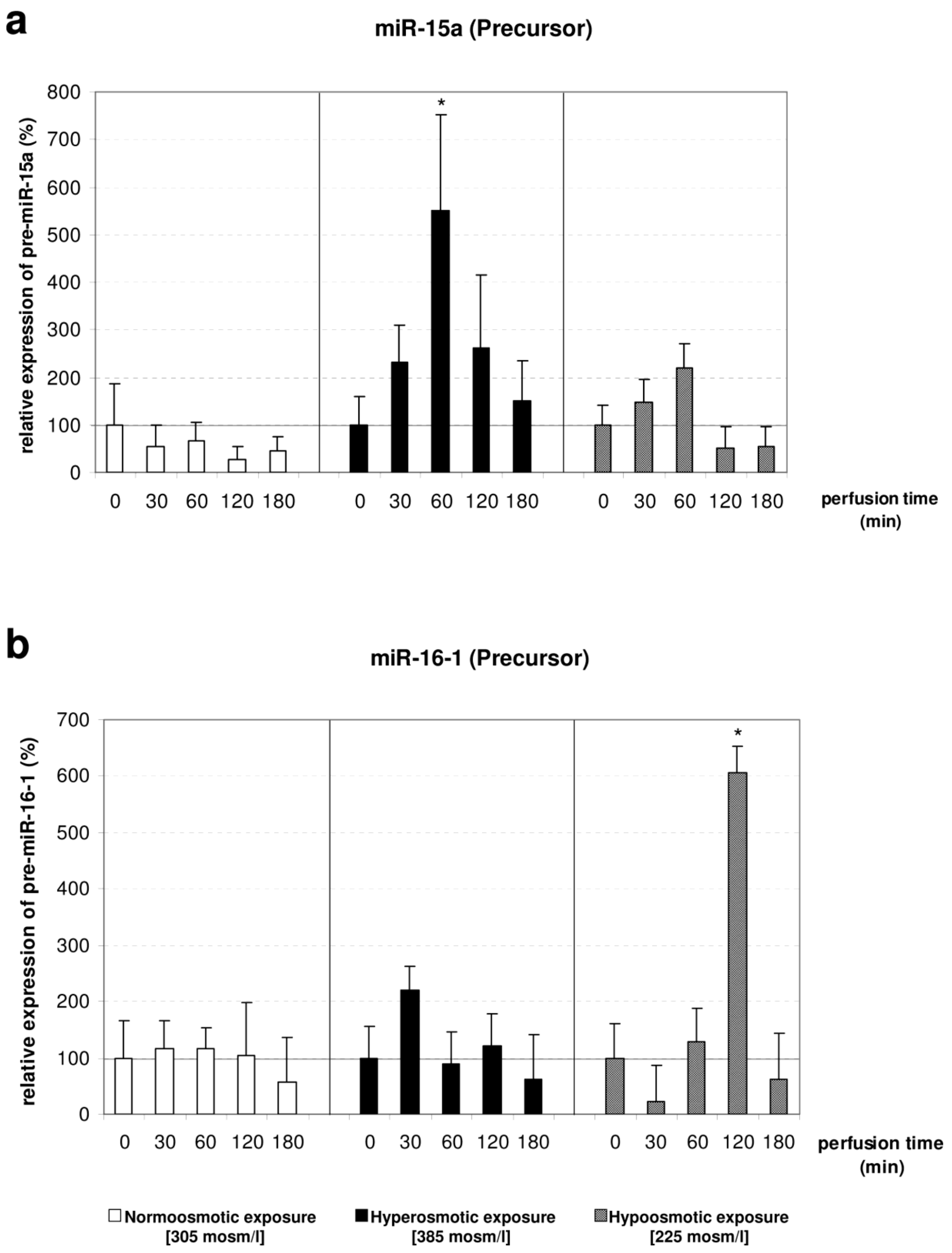

Supplementary Figure 4

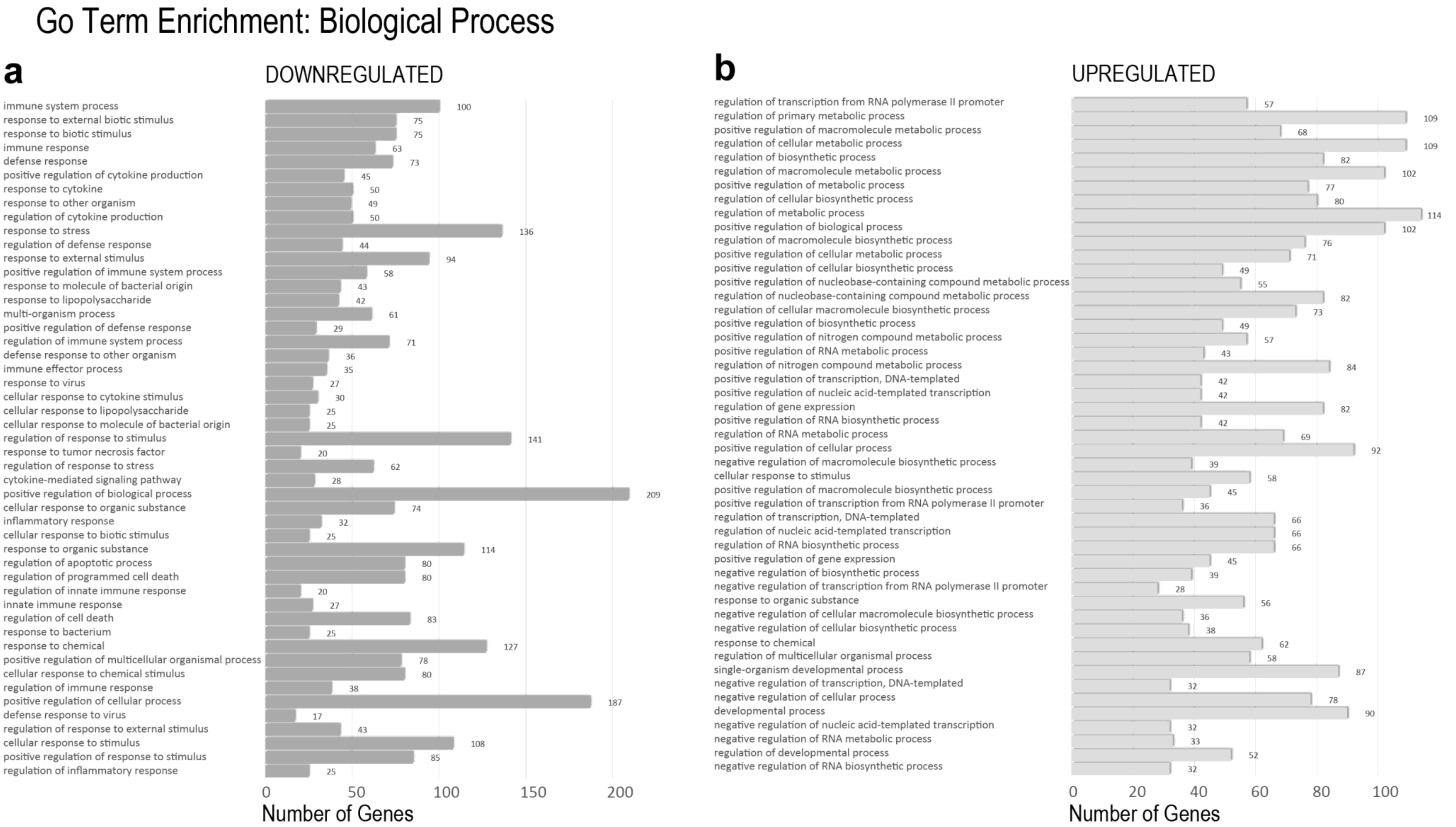

Supplementary Figure 5

Alignment between the sequence of conserved miR-15a/b/16 and the 3UTRs of putative targets

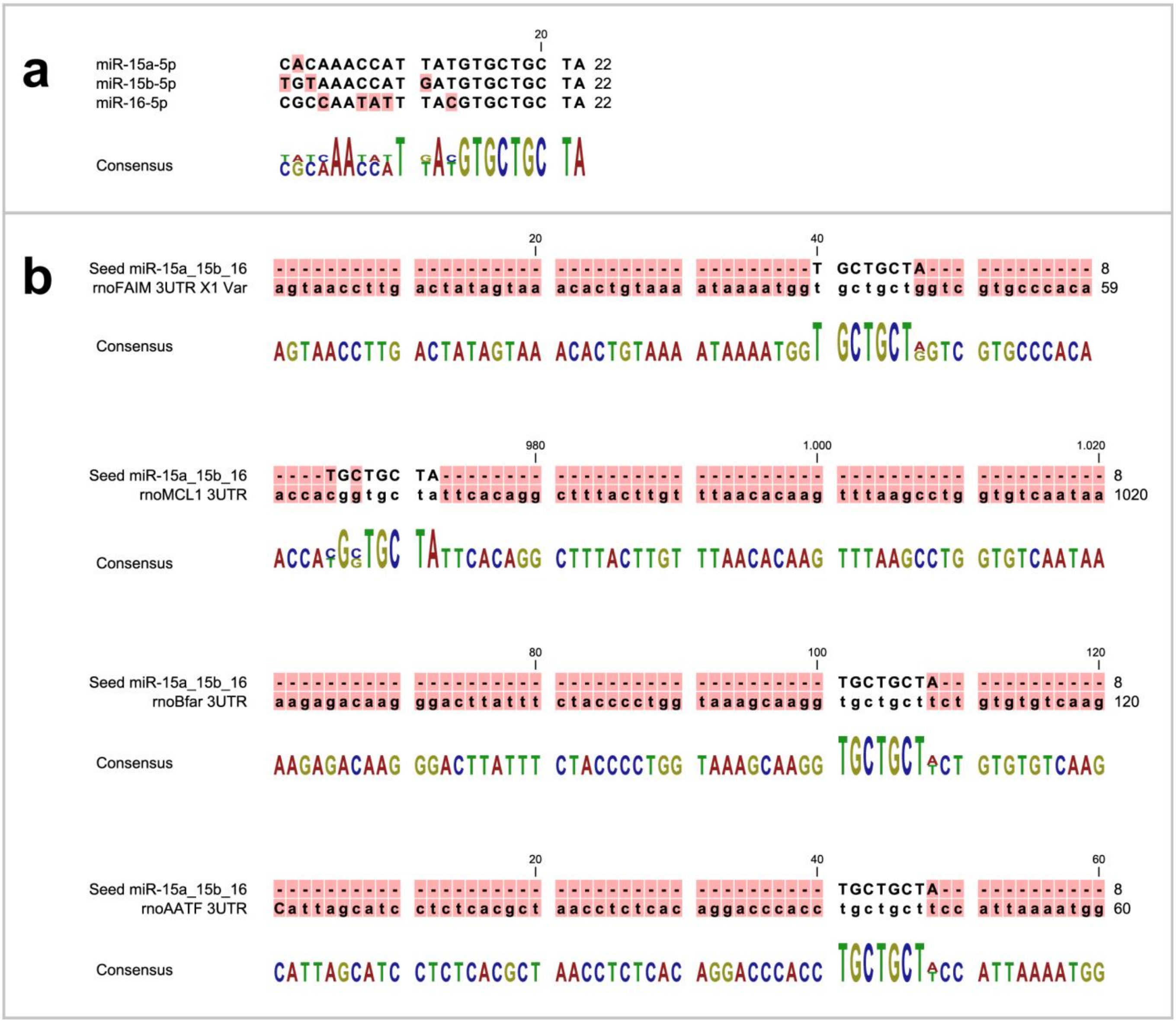

Supplementary figure 6

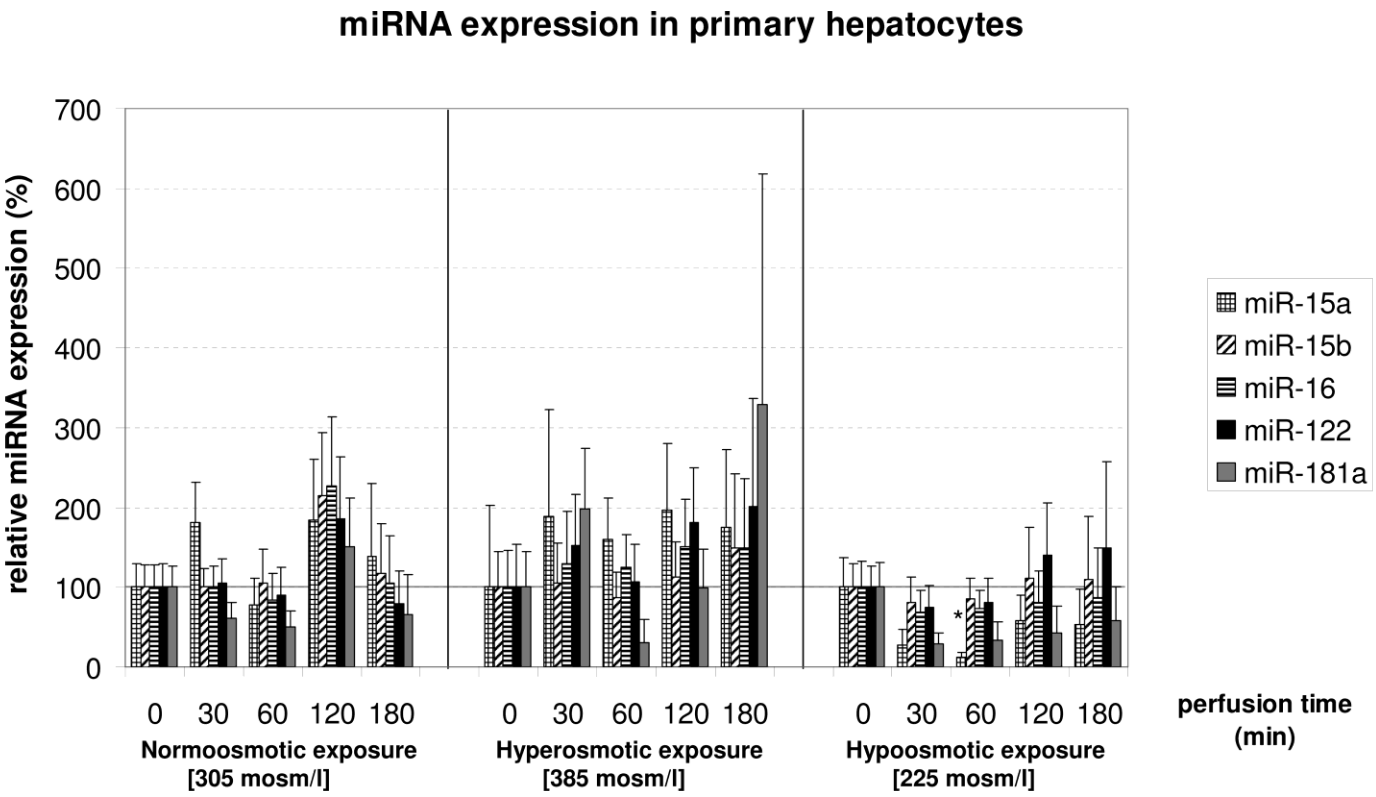

Supplementary Table 1

**Primers used for qPCR analysis of mRNA transcripts**	
**Name**	**Sequence**
rno-Aatf F	TGGACGAGGAGATATTTGATGA
rno-Aatf R	GCTGGTCTTCCGCTCTATGA
rno-Bcl2 F	GTACCTGAACCGGCATCTG
rno-Bcl2 R	GGGGCCATATAGTTCCACAA
rno-Bfar F	CGAGGACATCTTCACCAAGC
rno-Bfar R	GCTGATACGGGAGAAAGGAGT
rno-C3ar1 F	ATTTGTTGGTGGCTCACAGA
rno-C3ar1 R	TGTCAGCAGTGAAAGACTCCA
rno-Ccnd1 F	GCACAACGCACTTTCTTTCC
rno-Ccnd1 R	TCCAGAAGGGCTTCAATCTG
rno-Dcbld2 F	GGAATTGCTGGGATGTCAGT
rno-Dcbld2 R	GTGGGGGAGGCTGAGTAAG
rno-Faim F	TGGATGTATGGTGCAATGGT
rno-Faim R	GTCCCGTCATCTACAAACTCG
rno-Gapdh F	ACCACAGTCCATGCCATCAC
rno-Gapdh R	TCCACCACCCTGTTGCTGTA
rno-Gstm3 F	CCTTCCCAAACCTGAAGGAC
rno-Gstm3 R	GGGAGAAAGCTGCTACTCTTCA
rno-Ikbkb F	GAGAGCGTCAGCTGTGTCC
rno-Ikbkb R	CCCCACACTTTCCTCATCTG
rno-Irak2 F	GAGCTGCCCAGATTGTCCT
rno-Irak2 R	GCCGAGAGGGGAGACAAG
rno-Itgal F	TCTCCTTCCGAAAAGTGGAG
rno-Itgal R	CCTCGCAGCTCACAGGTATT
rno-Leprot F	TTGTTGTTTCTGCCTTTGGA
rno-Leprot R	AGCCAGCACAAGACCACAG
rno-Mcl1 F	AAAACTTTGTCATGATCAGTTCTAGTG
rno-Mcl1 R	TCTACTACAACCAGTCCACACACAC
rno-Nanp F	CCTGGACAACACACTCATCG
rno-Nanp R	TTGTAGTGGTATTTTGATTGTAAGAGC
rno-Nras F	AATACATGAGGACAGGCGAAG
rno-Nras R	TTTCACGCGCTTAATTTGC
rno-Oas1b F	AACACCCAGAGGTCTCCAAA
rno-Oas1b R	TCGAACCAGCAATGTTTCC
rno-Pcmt1 F	CCAGGTGGAAGTGATTTTCTCT
rno-Pcmt1 R	TCAGGTTGCTCTCACAGCTC
rno-Sema4a F	TGGCCCTGAATATCGAGAA
rno-Sema4a R	ACTGGCTGGCCAGGGTAT
rno-Slc11a2 F	CGGCCAGTGATGAGTGAGT
rno-Slc11a2 R	AGCAGACGAGAAGGACCAAG
rno-Slc16a6 F	GCCTTTTGGGGTTTATTCTCTT
rno-Slc16a6 R	CCTGGAACAAAGCTTCGAGT
rno-Tbp F	CCCACCAGCAGTTCAGTAGC
rno-Tbp R	CAATTCTGGGTTTGATCATTCTG
rno-Them4 F	TTGAGAAGCTGCGCTATGC
rno-Them4 R	CGACTTCCTCAGAAGAAAATAACC
rno-Tubb F	TCGTGGAATGGATCCCCAAC
rno-Tubb R	CTCCATCTCGTCCATGCCCT
rno-Zfp105 F	CGTGAGCATCTGTGGATCA

rno-Zfp105 R	CACTGCCATGTCCCAGAAC

Supplementary Table 2

**Primers used for qPCR analysis of miRNAs**	
**Name**	**Sequence**
pre-mmu-miR-15a	GCTGCCTCAAAATACAAGGAG
mmu-miR-15a	TAGCAGCACATAATGGTTTGTGG
rno-miR-15b	TAGCAGCACATCATGGTTTACAG
rno-miR-16	CAGCACGTAAATATTGGCGG
RNU6	GCAAGGATGACACGCAAATT
let-7a	TGAGGTAGTAGGTTGTATAGTTGG
rno-miR-21	TAGCTTATCAGACTGATGTTGAG
rno-miR-122	GAGTGTGACAATGGTGTTTGG
rno-miR-181a	AACGCTGTCGGTGAGTG

Supplementary figure and table legends

Supplementary Figure 1: PCMT is stably expressed following hyperosmotic liver perfusion, while it is upregulated under hypo- and normoosmotic exposure

PCMT1 is stably expressed under hyperosmotic stimulation, while in the other conditions it is significantly up-regulated. qPCR runs were normalized according to the ΔΔCt method using Gapdh as reference gene. Statistical analysis was carried out by unpaired student's t-test. Data are shown as average ± S.E.M. of 5 independent experiments.

Supplementary Figure 2: Expression of Bcl2 protein is stable until 180 minutes of hyperosmotic perfusion

(a) Rat livers were perfused with normoosmotic (305 mosmol/l) or hyperosmotic (385 mosmol/l) medium for the time period indicated. Bcl2 protein expression was analysed by Western blot and subsequent densitometric analysis. GAPDH served as a loading control. (**b)** Bcl2 shows a stable relative protein expression as assessed by densitometric analysis (no significant differences in both one-way ANOVA and unpaired student's t-test). Data are shown as average ± S.E.M. of 3 independent experiments.

Supplementary Figure 3: pre-miR-15a is significantly upregulated under hyperosmotic stimulation

(**a**) pre-miR-15a is significantly upregulated within 60 min of hyperosmotic perfusion, indicating osmoregulated changes on the transcriptional level. (**b)** pre-miR-16-1 is significantly upregulated under hypoosmotic stimulation at 120 minutes and is stably expressed under hyperosmotic conditions. qPCR runs were median normalized. Statistical analysis was carried out by unpaired student's t-test. Data are shown as average ± S.E.M. of 5 independent experiments.

Supplementary Figure 4: GO Term enrichment analysis for regulated genes on the Affymetrix data

GO Term enrichment analysis and ranked gene lists for biological processes were identified with GOrilla by using two unranked lists of genes with the whole Affymetrix Gene chip annotated to the rat genome as background list and (**a)** down- or (**b)** up-regulated genes as target lists.

Supplementary Figure 5: Putative binding positions of the miR-15a/b and miR-16 in 3'UTRs of predicted target genes

(a) Multiple sequence alignment of miR-15a, miR-15b and miR-16. **(b)** Bioinformatic analysis identified putative binding site for the miR-15a/b miR-16 seed sequence within the 3'UTRs of Faim, Mcl1, Bfar and Aatf

Supplementary Figure 6: Expression of selected miRNAs in primary rat hepatocytes

Analysis of miRNA expression in primary hepatocytes. miR-15a is significantly down-regulated under hypoosmotic exposure after 60 minutes. The other tested miRNAs are not significantly changed at any tested osmotic condition. qPCR runs were normalized according to the ΔΔCt method using let7a as reference gene. Statistical analysis was carried out by unpaired student's t-test. Data are shown as average ± S.E.M. of 5 independent experiments.

Supplementary Table 1: Primers for mRNA transcripts

Primers used for qPCR analysis of mRNA transcripts

Supplementary Table 2: Primers for miRNA analysis

Primers used for qPCR analysis of miRNAs
